# Retrieving zinc concentrations in topsoil with reflectance spectroscopy at Opencast Coal Mine sites

**DOI:** 10.1038/s41598-021-99106-1

**Published:** 2021-10-07

**Authors:** Bin Guo, Bo Zhang, Yi Su, Dingming Zhang, Yan Wang, Yi Bian, Liang Suo, Xianan Guo, Haorui Bai

**Affiliations:** College of Geomatics, Xi’an University of Science and Technology, Xi’an, China

**Keywords:** Environmental sciences, Environmental impact, Sustainability, Ecological modelling, Ecological modelling

## Abstract

Heavy metals contaminations in mining areas aroused wide concerns globally. Efficient evaluation of its pollution status is a basis for further soil reclamation. Visible and near–infrared reflectance (Vis–NIR) spectroscopy has been diffusely used for retrieving heavy metals concentrations. However, the reliability and feasibility of calibrated models were still doubtful. The present study estimated zinc (Zn) concentrations via the random forest (RF) and partial least squares regression (PLSR) using ground in-situ Zn concentrations as well as soil spectral reflectance at an Opencast Coal Mine of Ordos, China in February 2020. The coefficient of determination (R^2^), root mean square error (RMSE), mean absolute error (MAE), and the ratio of performance to deviation (RPD) were selected to assess the robustness of the methods in estimating Zn contents. Moreover, the characteristic bands were chosen by Pearson correlation analysis and Boruta Algorithm. Finally, the comparison between RF and PLSR combined with eight spectral reflectance transformation methods was conducted for four concentration groups to determine the optimal model. The results indicated that: (1) Zn contents represented a skewed distribution (coefficient of variation (CV) = 33%); (2) the spectral reflectance tended to decrease with the increase of Zn contents during 580–1850 nm based on Savitzky–Golay smoothing (SG); (3) the continuous wavelet transform (CWT) demonstrated higher effectiveness than other spectral reflectance transformation methods in enhancing spectral responses, the R^2^ between Zn contents and the soil spectral reflectance achieved the highest (R^2^ = 0.71) by using CWT; (4) the RF combined with CWT exhibited the best performance than other methods in the current study (R^2^ = 0.97, RPD = 3.39, RMSE = 1.05 mg kg^−1^, MAE = 0.79 mg kg^−1^). The current study supplied a scientific scheme and theoretical support for predicting heavy metals concentrations via the Vis–NIR spectral method in possible contaminated areas such as coal mines and metallic mineral deposit areas.

## Introduction

China is the world's largest coal producer and consumer, and coal provides more than 70 percent of total energy in China^1^. China has to confront the dilemma of balancing socio-economic development with environmental issues^2–7^. Exploring mineral resources may lead to adverse effects on the ecological environment^8,9^. Abundant heavy metals residues in tailings of open-pit mines have been generated due to inefficient processing procedures of ore. What's more, severe soil heavy metals contaminations have been caused in nearby farmland and urban areas because of the abandoned mine tailings that are exposed to the surrounding soils and rarely reclaimed^10–12^. Ordos Municipality, in the Inner Mongolia Autonomous Region of northern China, has been undergoing extensive opencast coal exploitation during past decades. The area of coal mining increased from 7.12 to 355.95 km^2^, and the number of coal-mining increased from 82 to 651 during 1990–2015 in the Ordos^13,14^. It’s reported that the Zn pollution in the topsoil of mining areas of Inner Mongolia and other places is relatively common^15,16^. Land productivity, ecological integrity, and habitat security were seriously threatened by heavy metals pollutions in mining areas^16–20^. Heavy metals are hazardous contaminants owing to their toxicity, persistency, easy uptake by plants, and long biological half-life^21,22^. Moreover, the heavy metals may cause stress on crops and hinder their growth, yield, and quality because the normal function of soil was destroyed by heavy metals^23,24^. Furthermore, human health is susceptible to heavy metals that may enter the body through biological chains^25,26^. If some heavy metals with both carcinogenic and teratogenic enter the bloodstream, they can dissolve red blood cells, destroy normal cells^27^. For example, Zn generates gastrointestinal distress including nausea, vomiting, and abdominal pain as well as irritation of the respiratory system^28^. Besides, cholesterol balance and fertility may be affected by Zn with long-term high dose exposure^29^. Lead (Pb) can lead to dysfunction in the immune system, the reproductive system, and hematopoiesis. Moreover, the brain, kidney, liver, and nerves may be damaged by the accumulation of Pb^30,31^. Cadmium (Cd) is one of the most toxicant hazardous materials, which can be absorbed by vegetables owing to its lipid. It can generate serious negative effects on public health with a level of > 0.2 mg·kg^−1^ in leafy vegetables. Additionally, Cd also blocks plant growth and photosynthesis of pigments^21^. Intaking of Nickel (Ni) may negatively impact public health owing to its accumulation. Previous studies have demonstrated an increased incidence of cancers to be related to chronic exposure to Ni^32^. Consequently, it is urgent to explore the distribution and evaluate the pollution level of heavy metals especially toxic materials such as Zn, Cd, and Pb in surface coal-mining areas^33,34^.

In-situ sampling and laboratory analysis are common methods with high accuracy for obtaining soil heavy metals contents^35^. The instruments including atomic absorption spectroscopy^36^, atomic fluorescence spectrometry^37^, spectrophotometry^38^, and other analytical methods based on optical instruments are always used to measure heavy metals concentrations. However, the above methods are time-consuming and costly^39,40^. Besides, it is inefficient to use the above methods to detect the spatiotemporal dynamic distribution of soil heavy metals on a large scale^41,42^. Alternatively, Vis–NIR spectroscopy with multiple bands (350–2500 nm), strong spectral continuity, and wide coverage, provides a new perspective for monitoring environmental issues over large scales^43^. Thus far, Vis–NIR spectroscopy has been widely utilized in different fields including predicting soil organic carbon^44^ and detecting heavy metals in agricultural soils, suburban soils, and river sediments^41,45,46^. Additionally, the mining areas accompany with complex topography increased the difficulty of exploring heavy metals distribution using traditional methods, which urged an alternative method for detecting hazardous materials in mining areas. The Vis–NIR spectroscopy supplied a new perspective to investigate heavy metals pollution. Currently, the accuracy of the calibration model for soil heavy metals concentration based on Vis–NIR spectroscopy is affected by many factors. Also, previous studies reported that spectral response information was hard to be extracted and stripped from weak soil spectral signals. The preprocessing of spectral reflectance can effectively promote the accuracy and robustness of the calibration model for heavy metals contents^47–49^. However, unsuitable preprocessing methods may lose the specific spectral information of toxic materials. CWT has been used to extract spectral detail information and proved it can effectively improve the prediction capacity of heavy metals concentrations using the Vis–NIR spectral inversion model^50^.

Moreover, the suitable calibration model using the Vis–NIR spectral method for heavy metals contents is very helpful. Published studies on Vis–NIR spectral inversion models for heavy metals concentrations can be divided into two classes including statistical analysis models and machine learning models^51,52^. Statistical analysis models including multiple linear regression (MLR), multiple linear stepwise regression (MLSR), principal component regression (PCR), and PLSR are widely used for determining heavy metals contents^53–55^. However, some issues such as the autocorrelation and multicollinearity of samples have been neglected when using linear regression to construct the models. Machine learning algorithms such as RF overcome the above problems, which linear or non-linear relationships between dependent and independent variables can be detected through the random forest^56–58^. Whereas, the related researches concerning the application of combining CWT with RF for estimating heavy metals concentrations in topsoil from coal-mining areas were still rarely reported. Thus, it is indispensable to compare the effects of different pretreatments on the calibration model and evaluate the efficiency of the spectral reflectance preprocessing techniques for determining the optimal preprocessing method in heavy metals concentration modeling. Meanwhile, the determination models of soil heavy metals concentrations were often performed using spectral variables from Vis–NIR spectral data collected from soil samples in the laboratory. However, spectra obtained in the laboratory and the field were completely diverse due to some uncertainties and disturbances including the preprocessing of samples, such as air-drying, grinding, and controlling the spectral measurement conditions in the laboratory. Soil spectra surveyed in the field were influenced by many factors such as soil particle size, soil surface conditions, soil water content, solar radiation, soil organic matter, temperature, and ambient light^21^. Therefore, it is still a big challenge to take advantage of the lab-derived models based on Vis–NIR spectroscopy to infer concentrations of heavy metals in soil. In general, the necessity of the current study was to evaluate the feasibility and reliability of using the Vis–NIR spectroscopy in estimating heavy metals contents at an open-pit coal mine, to compare the effect of various spectral transformation methods on the accuracy of the estimation models, and to determine if the concentrations of soil samples generate effects on the accuracy in retrieving heavy metals contents or not.

The objectives of this study are to (1) measure Zn concentrations, and survey in-situ reflectance spectra, lab-based processed reflectance spectra, and lab-based unprocessed reflectance spectra of soil samples from an Opencast Coal Mine of Ordos, China; (2) select optimal characteristic bands based on Pearson correlation coefficient as well as the Boruta algorithm; (3) calibrate Zn concentrations using statistical analysis and random forest based on Zn contents and spectral reflectance data; (4) evaluate the performance of related models including PLSR and RF combined with different spectral reflectance transformation methods, then determining the optimal prediction method for Zn contents.

## Materials and methods

### Study area

**Figure 1:**
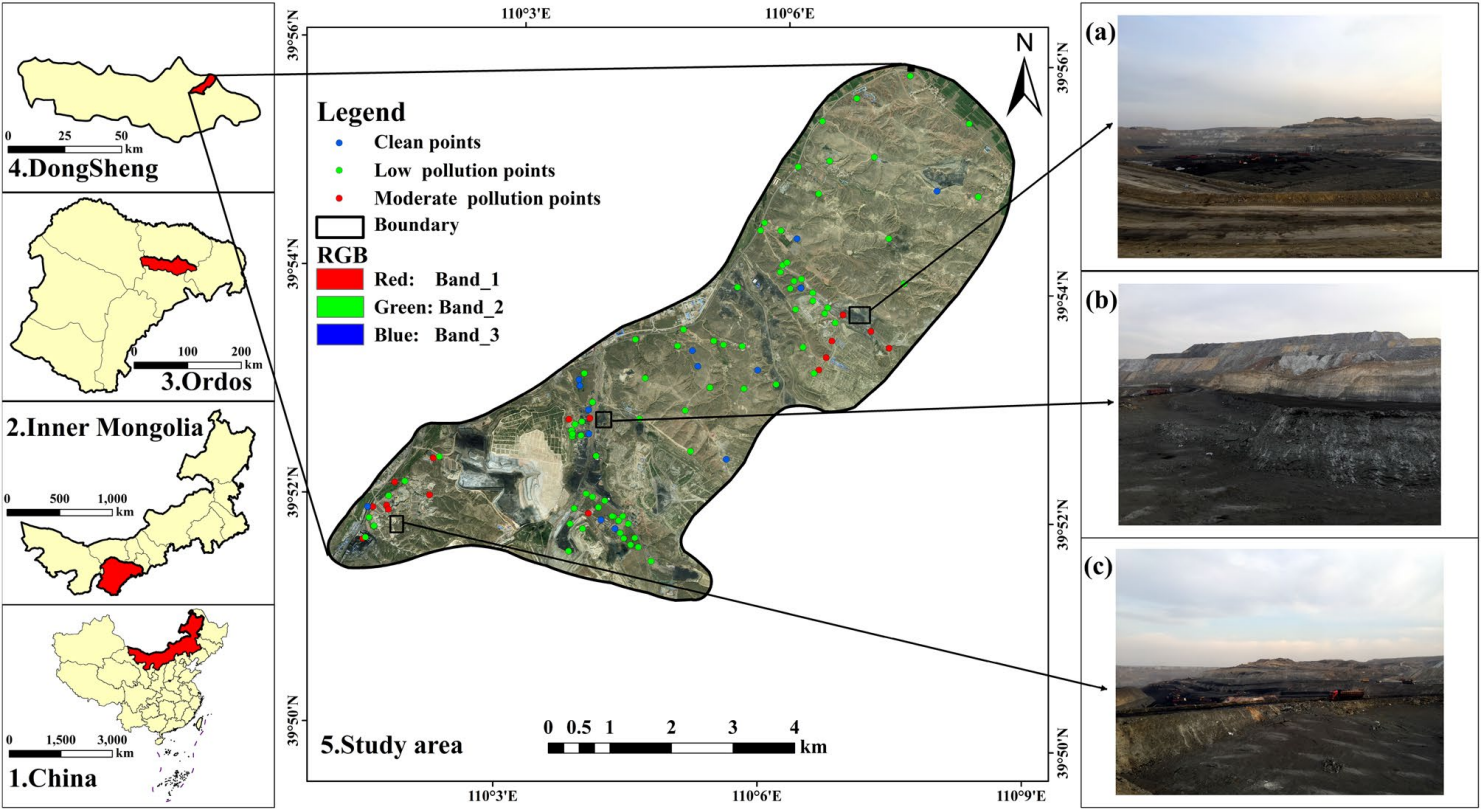
The geographical location of the study area and spatial distribution of the in-situ sampling sites are classified by pollution level. (**a**), (**b**), and (**c**) represent the actual conditions of the study area. (Note: soil samples were classified into three types based on the background value (BV)^15^ of the Inner Mongolia Autonomous Region using the contamination factor method^61,62^, including clean (Zn ≤ 48.6 mg kg^−1^), low pollution (48.6 ≤ Zn ≤ 97.2 mg kg^−1^), and moderate pollution (97.2 ≤ Zn ≤ 145.8 mg kg^−1^), respectively. The Fig. 1 was generated by ArCGIS 10.0 that was obtained from https://www.esri.com/en-us/arcgis/products/arcgis-desktop/, and the satelite image used in Fig. 1 (sub Fig. 5) was downloaded from Google Earth (https://earth.google.com/)).

The Ordos city, with an area of approximate 86,000 km^2^ and within 37°35′ ~ 40°51′ N and 106°42′ ~ 111°270′ E, is located in the Inner Mongolia Autonomous Region of north China. The topography with an elevation between 850 and 2149 m is high in the west and low in the east^59^. The Ordos city with a temperate continental climate has an annual sunshine duration between 2716.4 and 3193.9 h, an average annual temperature between 5.3 and 8.7 ℃, and mean annual precipitation ranging from 170 to 450 mm^14^. The Ordos has abundant coal resources. The coal-bearing area covers about 70% of the total area and the proven coal reserves account for 201.75 trillion tons. Specifically, the Ordos can be divided into four coalfields including the Zhungeer in the east, the Zhuozishan in the west, the Dongsheng in the south, and the Wulangeer in the north, respectively. Also, the Ordos has various types of coal, such as brown coal, cannel coal, and no-caking coal. Most of those coal resources are buried in a shallow layer that is suitable for opencast mining. Coal production in the Ordos increased from 6.11 million tons to 678.93 million tons from 1990 to 2019. The coal industries played an important role in the socio-economic development of Ordos. However, recently, the environment is deteriorating because of mining activities especially the seriously polluted soil (bare soil) near the mining areas. So, the present study chose the Dongsheng coalfield with an area of approximately 63.2 km^2^ as the sampling area^60^. The location of the study area and the distribution of the in-situ sampling sites are shown in Fig. 1.

### Workflow

**Figure 2:**
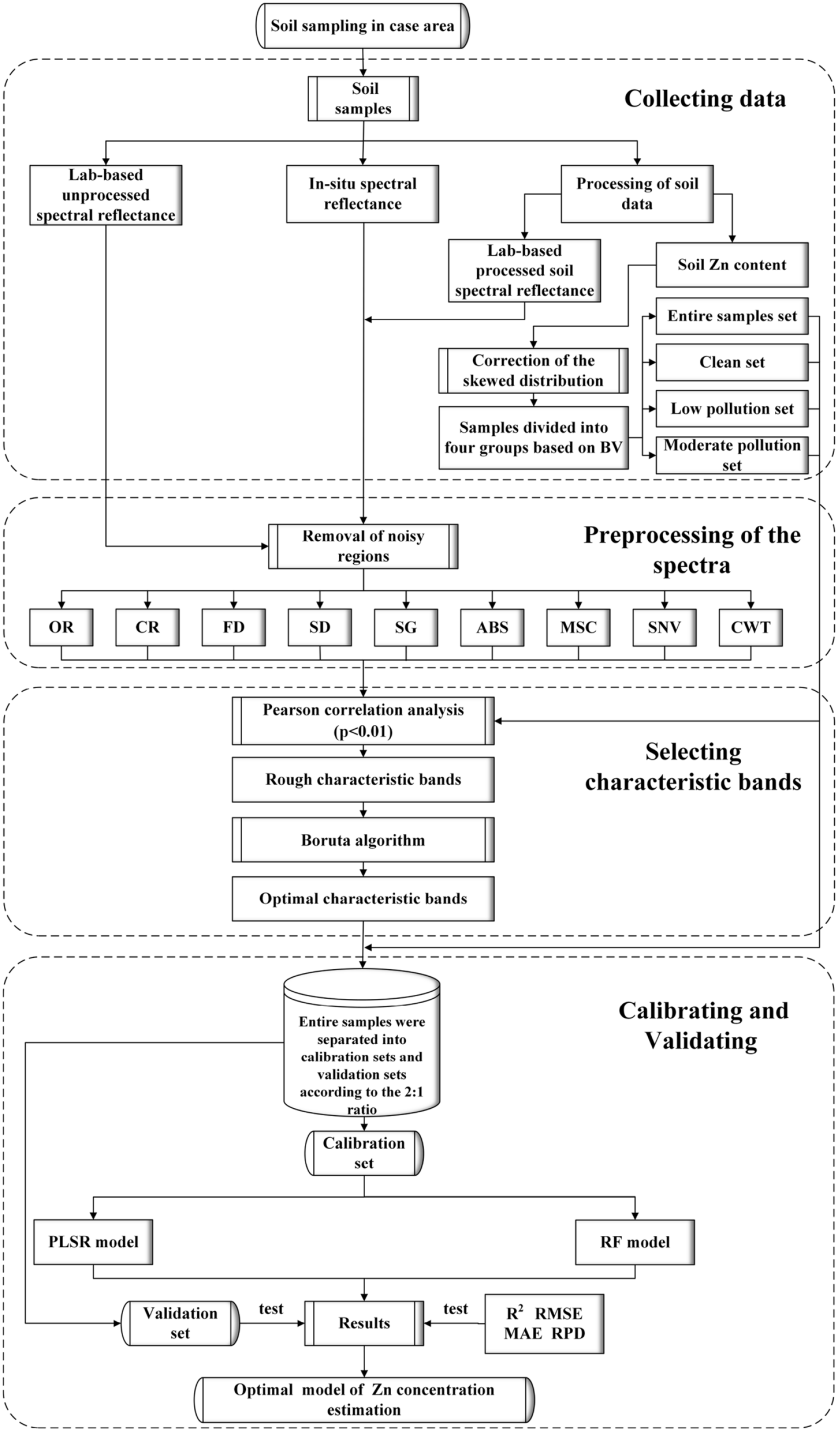
The workflow of this study. (Note: OR represents the original spectral reflectance of Zn).

The workflow of the current study was described as follows (Fig. 2): (1) Collecting three sorts of soil spectral reflectance including in-situ, lab-based processed, lab-based unprocessed, respectively, and the Zn concentrations were measured by an XRF instrument. A logarithmic transformation method was implemented to modify the skewed distribution of Zn concentration. Furthermore, soil samples were classified into three types based on the BV of the Inner Mongolia Autonomous Region using the contamination factor method, including clean (Zn ≤ 48.6 mg kg^−1^), low pollution (48.6 ≤ Zn ≤ 97.2 mg kg^−1^), and moderate pollution (97.2 ≤ Zn ≤ 145.8 mg kg^−1^), respectively. (2) Eight preprocessing methods in terms of continuum removal (CR), the first derivative of reflectance (FD), the second derivative of reflectance (SD), Savitzky–Golay smoothing (SG), absorbance transformation (ABS), Multiplicative Scatter Correction (MSC), Standard Normal Variate (SNV), and CWT were introduced to deduct spectral outliers and promote spectral response features of Zn after removing noisy regions (The detailed description of noisy regions can be found in “The spectral characteristics of soil samples under three conditions including in-situ, the lab-based processed, and the lab-based unprocessed”). (3) The integration of the Boruta algorithm and the Pearson correlation coefficients was adopted to choose the significantly important spectral variables for estimating Zn concentration. (4) The entire samples were separated into calibration sets and validation sets according to the 2:1 ratio. (5) PLSR and RF have been fitted to calibrate Zn concentration using the in-situ Zn concentrations dataset and the spectral reflectance of Zn, and the performance was compared based on specific indicators concerning R^2^, RMSE, MAE, and RPD.

### Sampling and measuring

The sampling route was designed according to FOREGS Geochemical Mapping Field Manual^63^ concerning agricultural production, industrial distribution, waste discharge, road and river networks, and soil type. A total of 111 in-situ soil samples (0–20 cm) were collected using a stainless-steel trowel in the study area on February 20, 2020. A portable Global Navigation Satellite System (GNSS) device was used to determine the World Geodetic System 1984 (WGS-84) coordinate of each sample site (Fig. 1)^64^. Each sample consisting of five subsamples with a wintersweet shape that were randomly collected from the surroundings, pooled, homogenized, and then reduced to a weight of 400 g to form a representative sample^23^. Additionally, samples were packaged back to the lab with plastic bags. In the lab, first, the samples were wind-dried. Then, soil samples were squashed with a glass stick and avoided impurities, crushed stone, and other alien elements. Next, an electric thermostatic air drying oven (DGG-9053AD, Shanghai, China) was utilized to exsiccate soil samples until constant weight. Next, all samples were sieved using a 0.7 mm polyethylene sieve and transferred into new plastic bags. Then each soil sample with 4 g weight was put into a 32 mm mold and squeezed a tablet with a boric acid edge under 30-ton pressure for X-ray fluorescence (SPECTRO xSORT, SPECTRO Analytical Instruments GmbH, Boschstr. 10, 47533 Kleve, Germany) analysis^65,66^. The mean content of every sample was determined by repeating three-time measurements for decreasing errors. Finally, specific software for X-ray fluorescence (SPECTRO xSORT) named Sample Result Manager was used to pretreat heavy metals concentrations data. The eight heavy metals concentrations including Zn, Cd, As, Co, Cu, Ni, Pb, and Mn were obtained. The GSD-series and GSS-series reference samples (Institute of Geophysical and Geochemical Prospecting, Lang fang, China) have been introduced to calibrate the SPECTRO xSORT, and the results demonstrated that the SPECTRO xSORT was reliable with a relative standard deviation ranged from 3 to 5%.

### Spectral measurements and preprocessing

The reflectance spectra of the soil samples were surveyed by a FieldSpec4 portable object spectrometer manufactured by ASD (Analytica Spectra Devices., Inc, USA) that covers 350–2500 nm spectral range with 1 nm spectral resolution. The reflectance spectral of three different types of samples including in-situ, lab-based processed, and lab-based unprocessed, respectively.

Under field conditions, the impurity such as stones, roots, leaves were excluded before sampling for assuring the purity of the soil samples. The soil spectra were measured using an ASD spectrometer at each position of five subsamples, and the mean spectrum of the five subsamples was chosen as the spectrum of the specific soil sample. To avoid the shadow when scanning the soil samples, firstly, making sure the probe was vertical to the ground. Secondly, adjusting the position to avoid the shadow to cover the soil samples, and to ensure the soil samples were completely exposed to the sun. Besides, a whiteboard with 99% reflectance was utilized to calibrate the spectrometer before measuring at each in-situ sampling site. Meanwhile, a warm-up with 30 min duration was carried out for the spectroradiometer to minimize errors. Additionally, 3-time spectral surveys were implemented, and calculated the mean value for each sampling site as the representative spectrum to decrease errors. All spectra were measured from 10 am to 2 pm under clear weather conditions because the sun was the only source of illumination at that condition. The fiber optic probe was put at approximately 15 cm above the soil samples vertically and in the opposite direction with solar radiation.

Accordingly, two kinds of samples concerning lab-based processed, and lab-based unprocessed reflectance spectra were surveyed under lab conditions using the same spectrometer. All spectral measurements were conducted in a dark room and all surveyors were required to dress in black clothes without any reflection to avoid unnecessary spectral noise. A 1000 W halogen lamp was used as the simulation light source. The field observation angle between the vertical direction and the light was set as 15°. The size of the soil samples container for spectra scanning is 10 cm × 10 cm. The field of view (FOV) is 25°, and the diameter of the field of view is 7 cm. Clearly, the surface area of the soil sample container completely covered the area of soil spectra. So, the size of the soil container can assure the purity of the soil samples spectra. The distance between the halogen lamp and the soil samples was 30 cm. Moreover, the distance and angle between the probe and the soil samples were 15 cm and 90°, respectively. The same whiteboard was also utilized to calibrate the spectroradiometer before measuring. Similar to the field spectral measurement, before starting the measurement, a warm-up of 30 min duration for the spectroradiometer was also implemented. Every sample was put into a black petri dish of a specific size. The size of the black petri dish for spectra scanning is 10 cm × 10 cm. The field of view (FOV) is 25°, and the diameter of the field of view is 7 cm. Clearly, the area of the soil sample container completely covered the area of soil spectra. The survey was conducted 3-time for reducing errors.

The process of collecting spectral reflectance was influenced by many potential factors such as survey device, soil sample, and lab conditions. The spectra may be worsened due to the above factors. So, the methods of transform spectral reflectance were always introduced to reduce the spectral noise. The Continuum Wavelet Transform (CWT) was selected to solve the above issue. Meanwhile, seven spectral transform methods including continuum removal (CR), the first derivative of reflectance (FD), the second derivative of reflectance (SD), Savitzky–Golay smoothing (SG), absorbance transformation (ABS), Multiplicative Scatter Correction (MSC), and Standard Normal Variate (SNV) were also implemented for comparison with the CWT. Twenty-one points and a quadratic polynomial were adopted to reduce spectral noise through the SG smoothing process.

Wavelet transform consists of two sorts concerning CWT and Discrete Wavelet Transform (DWT). The CWT was selected as a spectral transform method in this study. Spectral reflectance was decomposed into wavelet coefficients using different scales in terms of 2, 2^2^, 2^3^, 2^4^, 2^5^, 2^6^, 2^7^, 2^8^, 2^9^, 2^10^ (L_1_–L_10_) based on Gaussian4 function served as the mother wavelet^50^. The Gaussian4 was adopted as the mother wavelet function for soil spectral absorption features was close to the Gaussian function^67^. The basic function of the Wavelet Transform was as follows.

If $\psi \left( t \right) \in L^{2} \left( R \right)$ is a square-integrable function of its Fourier transform, it then satisfies the following expression:

1 $$\begin{array}{*{20}c} {C_{\psi } = \mathop \int \nolimits_{R} \frac{{\left| {\psi^{\Lambda } \left( \omega \right)} \right|^{2} }}{\left( \omega \right)}{\text{d}}\omega < \infty } \\ \end{array}$$

where $\psi \left( t \right)$ denotes the wavelet basis function.

The wavelet basis function can be scaled and assessed to obtain the wavelet basis function,$\psi_{\alpha ,\tau } \left( t \right)$, as follows:

2 $$\begin{array}{*{20}c} {\psi_{\alpha ,\tau } \left( t \right) = \frac{1}{\sqrt \alpha }\psi \left( {\frac{t - \tau }{a}} \right) \alpha ,\quad \tau \in R; \alpha > 0} \\ \end{array}$$

where $a$ denotes the scale factor, $\tau$ denotes the translation factor, and $t$ denotes the spectral bands. One-dimensional spectra were transformed into a two-dimensional m $\times$ n matrix by CWT. Each row of the matrix denotes a wavelet coefficient at a different decomposed scale.

### Spectral feature selection and correction of skewed data

#### Spectral feature selection

The Boruta algorithm^68,69^ designed as a wrapper around a Random Forest classification was introduced to conduct the spectral feature selection because it can provide an intrinsic measure of the importance of each variable, called the Z-score. The Z-score of the original variables and the expected Z-score from the randomly selected features generated by random permutation were compared to determine the terminal variables with a larger Z-score than that of all the randomly selected features. In this study, the integration of the Boruta algorithm and the Pearson correlation coefficients with a significance level of 0.01 was used to select the significantly important spectral variables for estimation of the Zn concentration^70^.

#### Correction of skewed data

Logarithmic transformations are widely used to adjust a highly skewed variable into a more approximately normal variable due to their effectiveness and convenience^64^. Besides, logarithmic transformations are always used in conditions where the independent and dependent variables exhibit a nonlinear relationship and still preserve the linear regression model. Specifically, soil samples generally represent lognormal distributions^71^. So, the natural logarithm transformation was selected to correct the skewed distributions of the heavy metals concentration in the current study.

### Calibration and validation

#### Partial least squares regression (PLSR)

PLSR proposed by Herman O. A. Wold is a spectral analysis method that includes multiple linear regression, canonical correlation analysis, and principal factor analysis^72^. PLSR is suitable for Vis–NIR spectral bands with collinearity and spectral noise^73^. PLSR projects a group of spectral and dependent response variables into a low-dimensional space, thereby decreasing dimensionality and excluding noise. Recently, PLSR has been widely used in soil heavy metals concentrations retrieving based on Vis–NIR spectral technology^74^. Leave-one-out cross-validation was introduced to obtain the number of latent variables (LVs) of PLSR. The maximum number of LVs was chosen to control the number of LVs to eliminate over-fitting. The number of LVs with the lowest root mean square error of cross-validation (RMSECV) was adopted in the calibration^37^.

#### Random forest (RF)

The RF algorithm^75^ is a bagging method based on regression tree (CART) analysis and classification^76^. The advantages of RF are the significance of each feature can be assessed with unbiased estimation during the classification process, and the issues with numerous missing data can be solved. Additionally, the efficiency of the RF model in processing big data without any dimensionality reduction outperforms traditional models^77^. The classification trees are used to decide on choosing the optimal tree in predicting. The number of classification trees in RF is large, and all variables have to be inputted into each tree with an independent feature for classing. Moreover, 99.9% of unrelated trees conduct predictions that cover all conditions. The basic theory of RF bagging is to choose the results of several weak classifiers and form a strong classifier. The processes for generating classification trees and mathematical equations of the RF model can be found in related literature, the current study did not state corresponding contents again due to limited space^78,79^. Three parameters including the number of trees of the classification tree (ntree), the variable selection number (mtry) when branching, and the size of leaf (node size) were important for constructing an RF model. The default parameters for the node size were adopted to construct each model^67^. Several parameters were tested for determining the optimal value of mtry and ntree of the RF model. So, the optimization of mtry ranged from 1 to 100 at the interval of 1, and the best ntree varied from 1 to P−1 at the interval of 1. P represents independent variables.

#### Validation

Before the calibrating, the samples needed to be grouped. The entire samples were separated into calibration sets and validation sets according to the 2:1 ratio. The concentrations of heavy metals were ranked from the lowest to the highest for selecting three adjacent samples as a group. For each group, two samples were chosen randomly as the calibration set, and the remained one was selected as the validation set^80^. The calibration set was used to fit the model, and the validation set was utilized to assess the performance of the model.

Four indicators including coefficient of determination (R^2^), root mean squared error of prediction (RMSE), mean absolute error (MAE), and the ratio of performance to deviation (RPD) was chosen for evaluating the accuracy and robustness of the models. The detailed information concerning the four accuracy indicators was not described due to the limited space, and the related statements can be found in previous studies^81–84^.

### Software

Spectral pretreatments and the Boruta algorithm were executed via R version 3.6.3. Moreover, the PLSR and RF models were run in MATLAB version 2016b. Finally, Sample Result Manager (a specific software for SPECTRO xSORT), and ArcGIS10.0 were used for analyzing and mapping in this paper.

## Results

### Descriptive statistic

**Figure 3:**
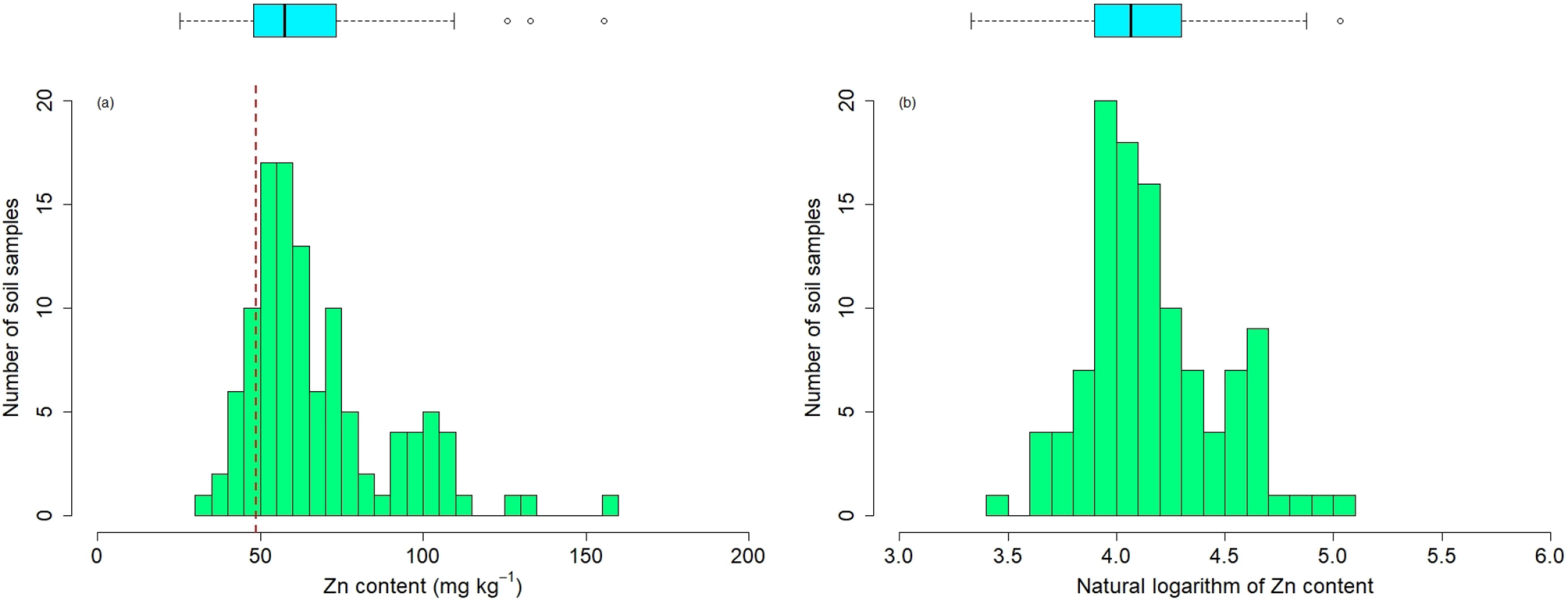
Histogram and box plot of Zn concentration of the topsoil in the study area (No. of samples = 111). (Note: Rad dashed curve is the soil Zn concentration background value of Inner Mongolia Autonomous Region, dashed circle denotes the outliers of soil Zn concentration).

Table 1 and Fig. 3a revealed the statistical features of the Zn element. The concentration of Zn ranged from 30.05 to 157.00 mg kg^−1^. Although the mean concentration of Zn (67.98 mg kg^−1^) did not surpass the background values of the Chinese Environment Quality Standard for Soils released by the Ministry of Environmental Protection of China in 2018 (200 mg kg^−1^) (GB15618-2018), it exceeded the background value of the Inner Mongolia Autonomous Region (48.6 mg kg^−1^)^15^. Moreover, more than 87% of the soil samples were polluted by the Zn element, 73% and 14% account for minor pollution as well as moderate pollution (Fig. 1). The outcome demonstrated that plenty of Zn deposited in the topsoil of the study area. Also, the SD (22.48 mg kg^−1^) of Zn concentration was relatively high because some samples exhibited extremely higher concentrations (157, 135, and 128 mg kg^−1^) than the neighbors. Additionally, the variation of the measured Zn concentration demonstrated significant spatial heterogeneity based on the range (30.05 to 157.00 mg kg^−1^), SD (22.48 mg kg^−1^), and CV (33%) for Zn. We can infer that the concentration of Zn in the topsoil of the study area was largely affected by long-term anthropic activities especially mining activities. The Table1 showed that Zn represented a skewness distribution (Skewness = 1.29) with heavy tails (Kurtosis = 1.68). The skewed and irregular distribution may generate negative effects on retrieving soil metals contents with Vis–NIR spectroscopy^64,85^. Therefore, a natural logarithm transformation was selected to correct the negatively skewed distribution (Fig. 3b).

**Table 1:** Statistics of the collected soil samples for Zn concentration (mg kg^−1^).

Element	Maximum	Minimum	Mean	Range	Median	SD^a^	CV^b^	Skewness	Kurtosis	BV^c^	PR^d^ %
Zn	157.00	30.05	67.98	126.95	61.30	22.48	33	1.29	1.68	48.6	87.39

^a^SD: standard deviation, ^b^CV: coefficient of variation in %.

^c^BV: the soil Zn concentration background value of Inner Mongolia Autonomous Region^15^.

^d^PR: the percentage of contaminated samples (Threshold = 48.6 mg kg^−1^).

### The spectral characteristics of soil samples under three conditions including in-situ, the lab-based processed, and the lab-based unprocessed

The in-situ soil spectra from the mining area were shown in Fig. S1e. The lab-based processed spectra and the lab-based unprocessed spectra from the mining area were illustrated in Fig. S1a,c), respectively. Clearly, though the spectral reflectance of each soil sample changed according to the wavelength, the varied trend of spectral reflectance for all soil samples was similar. The spectral curves with smoothed features represented an upward trend, and the spectral reflectance values were ranged from 0 to 0.6 (Fig. S1a). Specifically, the curves were separated into three wavelength bands. Although spectral reflectance was low, the values increased rapidly during the visible light band (400–780 nm). The spectral reflectance was relatively stable and high at the short-wave near-infrared waveband (780–2100 nm). The spectral reflectance decreased slowly during the long-wave near-infrared waveband (2100–2500 nm). Clearly, atmospheric water vapor exhibited strong absorptive effects on spectra especially around 1400 and 1900 nm and above 2400 nm during field spectral measurement^86^. So, the spectral noise aroused by atmospheric water vapor has been removed from the raw spectra (Fig. S1e,f), and the spectra mainly distributed during 350 –399 and 2400–2500 nm defined as noisy regions where reflectance spectra exhibited unstable features and were always excluded in published studies has been removed (Fig. S1b,d)^87^. Another obvious fluctuation of the curves around 1000 nm was detected probably due to the interference of iron oxide^88^. A strong absorptive belt of the spectral curves was found at the near-infrared region. Specifically, water led to the obvious absorption at 1400 nm and 1900 nm, and the crystal lattice water at 1450 nm and 2200 nm also presented significantly absorptive capabilities (Fig. S1b,d)^37^. The spectral reflectance increased sharply from 400 nm due to the presence of organic matter and iron ions^89^. Meanwhile, one small valley occurred at approximately 2200 nm owing to metals hydroxyl stretching^45,90^. Obviously, the spectral reflectance obtained from the field was relatively lower than collected from the laboratory because the soil water exhibits an absorption effect on spectral reflectance (Fig. S1a,e).

The mean CR, FD, SD, SG, ABS, MSC, SNV, and CWT spectral reflectance curves under lab-based processed are illustrated in Fig. S2. The same methods were implemented for the other two situations including lab-based unprocessed and in-situ, and the results were presented in Figs. S3 and S4. Clearly, the reflectance spectra of soil were decreased with the concentration increased during the wavelengths 580–850 nm (Fig. S2SG, MSC). The reflectance spectra showed similar spectral shapes but with variable spectral intensities because the color of the soil sample gradually darkened may be affected by the increase in the heavy metals content, and the reflectance absorbed additional light energy, such that the spectral curve slowly decreased^91^. CWT could hardly extract obvious spectral response at the L_1_–L_3_ scales (Fig. S2L_1_–L_3_). On the contrary, the relatively significant spectral response with sharp absorption peaks could be retrieved in the condition of increasing of decomposition scales (Fig. S2L_4_–L_6_). Meanwhile, the spectral strength gradually was enhanced with the CWT scales increasing (Fig. S2L_1_–L_10_). FD, SD, ABS, SG, MSC, and CR revealed a relatively weak capacity in increasing the responses for reflectance spectra compared with CWT in a particular wavelength. The original spectral reflectance curve generated by absorption was relatively less pronounced with broad and smooth features. The absorption peaks appeared at approximately 1400, 1900, and 2200 nm. So, the spectral reflectance transformation methods were conducted to enhance the original spectral reflectance response. The characteristics of the original spectral reflectance have been increased. Absorption peaks were observed at approximately 500, 950, 1350, 1900, and 2200 nm using CR, 550, 1000, 1325, 1350, 1375, 1800, 1875, 2200, and 2250 nm via FD, and 1000, 1375, 1800 nm through SD. The baseline drifts and mixed overlapping peaks were efficiently deducted by FD and SD because the spectral reflectance became gradually approximately 0.

### Selecting the characteristic bands based on Pearson correlation coefficients and the Boruta algorithm

First, the Pearson correlation coefficients were implemented to analyze the relations of Zn concentration for the three groups corrected by the natural logarithmic transformation method with soil spectral reflectance transformed by eight methods including CR, FD, SD, SG, ABS, MSC, SNV, and CWT. The rough characteristic bands were determined by the correlation coefficients square (R > 0.6)^50,92^. Second, The Boruta algorithm was carried out to choose the optimum characteristic bands based on the Z-score using the rough characteristic bands.

**Figure 4:**
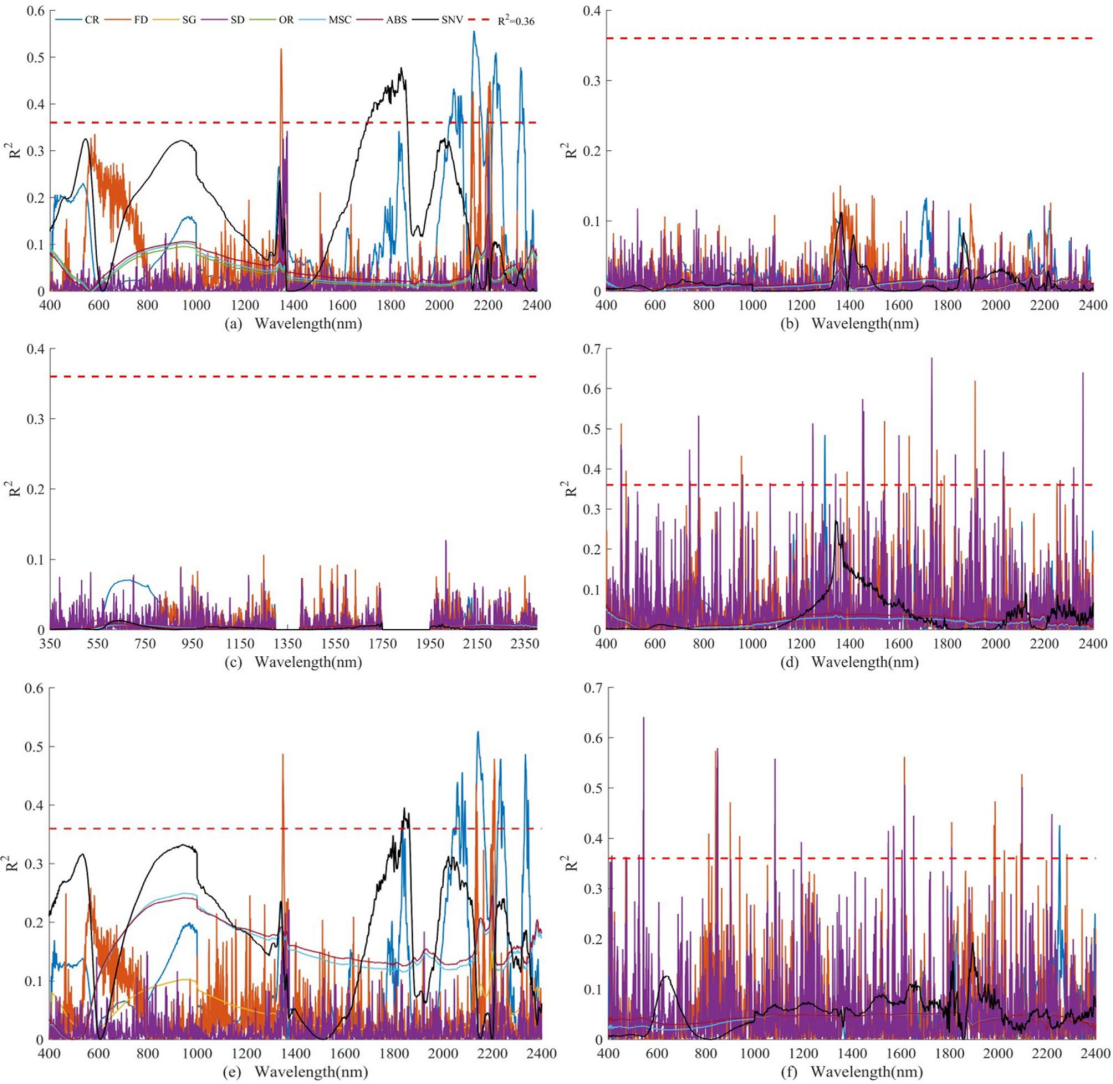
Correlation of determination (R^2^, P < 0.01) between Zn concentrations corrected by natural logarithmic transformation and spectral reflectance transformed by CR, FD, SG, SD, MSC, ABS, and SNV under six conditions of soil samples, including (**a**): lab-based processed, (**b**): lab-based unprocessed, (**c**): in-situ, (**d**): clean group (Zn ≤ 48.6 mg kg^−1^), (**e**): low pollution group (48.6 ≤ Zn ≤ 97.2 mg kg^−1^), and (**f**): moderate pollution group (97.2 ≤ Zn ≤ 145.8 mg kg^−1^). (Note: The spectral reflectance was measured after processing at the laboratory for the clean group, low pollution group, and moderate pollution group.)

Figure 4 revealed that all of the R^2^ for the original spectrum was less than 0.1 and below the red dashed line (R^2^ = 0.36). The R^2^ of the spectrum after transforming was notably larger than the original spectrum that represented the spectral reflectance transformation methods could increase the sensitivity of reflectance response sheltered in the soil spectral reflectance data compared to the original spectral variables. The peak positions for R^2^ varied according to the spectral transformation methods and spectral wavelength. For the two conditions without any sample processing in terms of lab-based unprocessed and in-situ, no matter what spectral reflectance transformation methods we chose no obvious rough characteristic bands were existing. On the contrary, the rough characteristic bands were mainly concentrated during about 1347–1354 nm, 1699–1867 nm, 2041– 2096 nm, 2132–2174 nm, 2196–2210 nm, 2218–2251 nm, 2330–2347 nm for lab-based processed. Meanwhile, three transformation methods including CR, FD, and SNV exhibited higher sensitivity than other methods for detecting rough characteristic bands. The maximum R^2^ = 0.56 was found at 2142 nm with CR, followed by 1349 nm with an R^2^ = 0.52 of FD, and 1843 nm with an R^2^ = 0.48 of SNV (Fig. 4a). For the three group concentration concerning clean, low pollution, and moderate pollution corrected by the natural logarithmic transformation method, the number of rough characteristic bands of the low pollution group was the largest (Fig. 4e), followed by the clean group (Fig. 4d), and moderate pollution group (Fig. 4f). The rough characteristic bands were listed in Table S1. Besides, three transformation methods including CR, FD, and SNV revealed higher sensitivity than other methods for detecting rough characteristic bands for the low pollution group. CR, FD, and SD demonstrated higher sensitivity than other methods for detecting rough characteristic bands for the clean and moderate pollution group. For the clean group, the largest R^2^ was found at 1298 (R^2^ = 0.48), 1915 (R^2^ = 0.61), and 1737 (R^2^ = 0.68) nm using CR, FD, and SD, respectively (Fig. 4d). For the low pollution group, the maximum R^2^ was found at 2142 nm with an R^2^ of 0.53 (CR), 1349 nm with an R^2^ of 0.48 (FD), and 1842 nm with an R^2^ of 0.40 (SNV), respectively (Fig. 4e). For the moderate pollution group with the SD transformation method, the largest value was detected at 545 nm with R^2^ of 0.64. Six spectral positions including 2098 nm, 1616 nm, 846 nm, 849 nm, 1084 nm, and 848 nm appeared peak values ranging from 0.50 to 0.58 (Fig. 4f). For moderate pollution group with other transformation methods (Fig. 4f).

**Figure 5:**
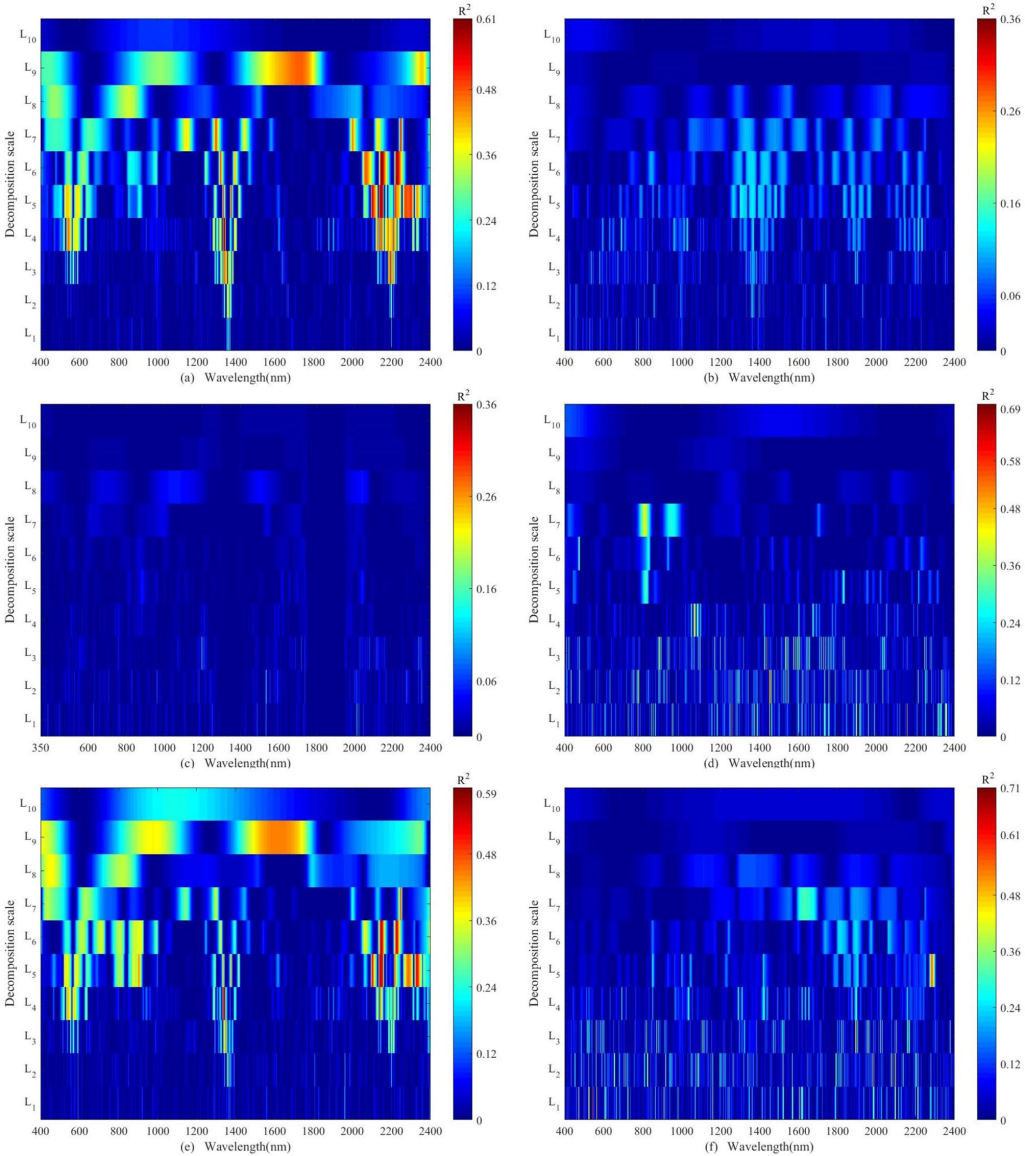
Correlation of determination (R^2^, P < 0.01) between Zn concentrations corrected by natural logarithmic transformation and spectral reflectance transformed by CWT under six conditions of soil samples, including (**a**):lab-based processed, (**b**): lab-based processed, (**c**): in-situ, (**d**): clean group (Zn ≤ 48.6 mg kg^−1^), (**e**): low pollution group (48.6 ≤ Zn ≤ 97.2 mg kg^−1^), and (**f**): moderate pollution group (97.2 ≤ Zn ≤ 145.8 mg kg^−1^). (Note: The spectral reflectance was measured after processing at the laboratory for the clean group, low pollution group, and moderate pollution group. L_1_–L_10_ denotes the reconstructed spectral reflectance curves based on CWT at decomposition scales of 1–10.)

The CWT was implemented on the raw spectral reflectance for spectral transformation, and the Gaussian4 function was selected as the wavelet basis function and the decomposition scales were divided into 10 scales including 2, 2^2^, 2^3^, 2^4^, 2^5^, 2^6^, 2^7^, 2^8^, 2^9^, 2^10^ (L_1_–L_10_). Overall, the R^2^ values and the number of the rough characteristic bands varied accordingly among the different wavelet decomposition scales. Specifically, the number of the rough characteristic bands was increased with the increasing wavelet decomposition scales excluding L_7_ and L_8,_ especially for the lab-based processed situation. For lab-based processed situations and low pollution group, the optimal decomposition scales were L_4_, L_5_, L_6_, L_7_, and L_9_ according to the R^2^ values. The rough characteristic bands were listed in Table S2 and were highlighted in red in Fig. 5a and Fig. 5e. On the contrary, for the other four situations including lab-based unprocessed, in-situ, the clean group, and moderate pollution group, the rough characteristic bands were more scattered and less obvious. (Fig. 5b,c,d,f). Moreover, the rough characteristic bands were completely not detected under the conditions of lab-based unprocessed as well as in-situ (Fig. 5b,c).

For the clean group, the rough characteristic bands were located at positions L_1_, L_2_, and L_3_ (Fig. 5d). Specifically, on decomposition scale L_1_, the rough characteristic bands occurred at 1736, 1242, 744, 1921, 742, 1259, 2158, 1501, 480, and 2319 nm. On decomposition scale L_2_, the rough characteristic bands appeared at 1456, 778, 1541, 2032, 774, and 1546 nm. On decomposition scale L_3_, the rough characteristic bands appeared at 1343 nm. For the moderate pollution group, the rough characteristic bands were situated at the four decomposition scales of L_1_, L_2_, L_3_, and L_5_ (Fig. 5f). On decomposition scale L_1_, the rough characteristic bands appeared at 901, 544, 903, 1616, and 1807 nm. On decomposition scale L_2_, the rough characteristic bands appeared at 1985, 2094, and 2095 nm. On decomposition scale L_3_, the rough characteristic bands appeared at 1895, 1896, and 2102 nm. On decomposition scale L_5_, the rough characteristic bands appeared at 2080–2090 nm.

The Boruta algorithm was carried out to determine the optimum characteristic bands for the prediction of Zn concentrations based on the rough characteristics bands (R^2^ > 0.36) obtained by Pearson correlation analysis. The important Z-score calculated by the Boruta algorithm of rough characteristics bands were used to choose the optimum characteristic bands after lab-based processed for the entire group (Fig. S5), clean group (Fig. S6), the low pollution group (Fig. S7), and the moderate pollution group (Fig. S8).

Clearly, the position and the number of optimal characteristics bands highlighted in green dots changed with transformation methods (Fig. S5). Forty-nine optimal characteristics bands were selected through CR spectral transformation (Fig. S5CR). It was found that 22 spectral variables were important for Zn estimation after the FD spectral transformation, and 1 spectral variable was excluded. Also, 25 characteristic bands were important for predicting Zn content by SNV spectral transformation, and the other 139 bands could be ignored due to the lower Z-score. Meanwhile, there were 6, 18, 26, 28, 46, 47, 45 characteristics bands for L_2_, L_3_, L_4_, L_5_, L_6_, L_7_, and L_9_ respectively through CWT spectral transformation. Then, the optimal characteristics bands were inputted into calibration models as independent variables for estimating Zn concentration.

### Calibrating and comparing Zn concentration based on the PLSR and RF models using optimal characteristic bands of each spectral reflectance transformation method for four sorts of Zn concentration groups including clean, low pollution, moderate pollution, and the entire samples, respectively

The in-situ and lab-based unprocessed situations were excluded for no characteristic bands were detected according to Sect. 3.3 (Figs. 4, 5). So, the optimal characteristic bands determined by the Boruta algorithm for lab-based processed soil samples were used to calibrate the Zn concentration corrected by natural logarithmic transformation using RF and PLSR for four groups concerning the entire samples, clean group, low pollution group, and the moderate pollution group. The calibration results for spectral reflectance transformation methods combined with RF and PLSR were compared and evaluated by R^2^, MAE, RMSE, RPD respectively.

**Figure 6:**
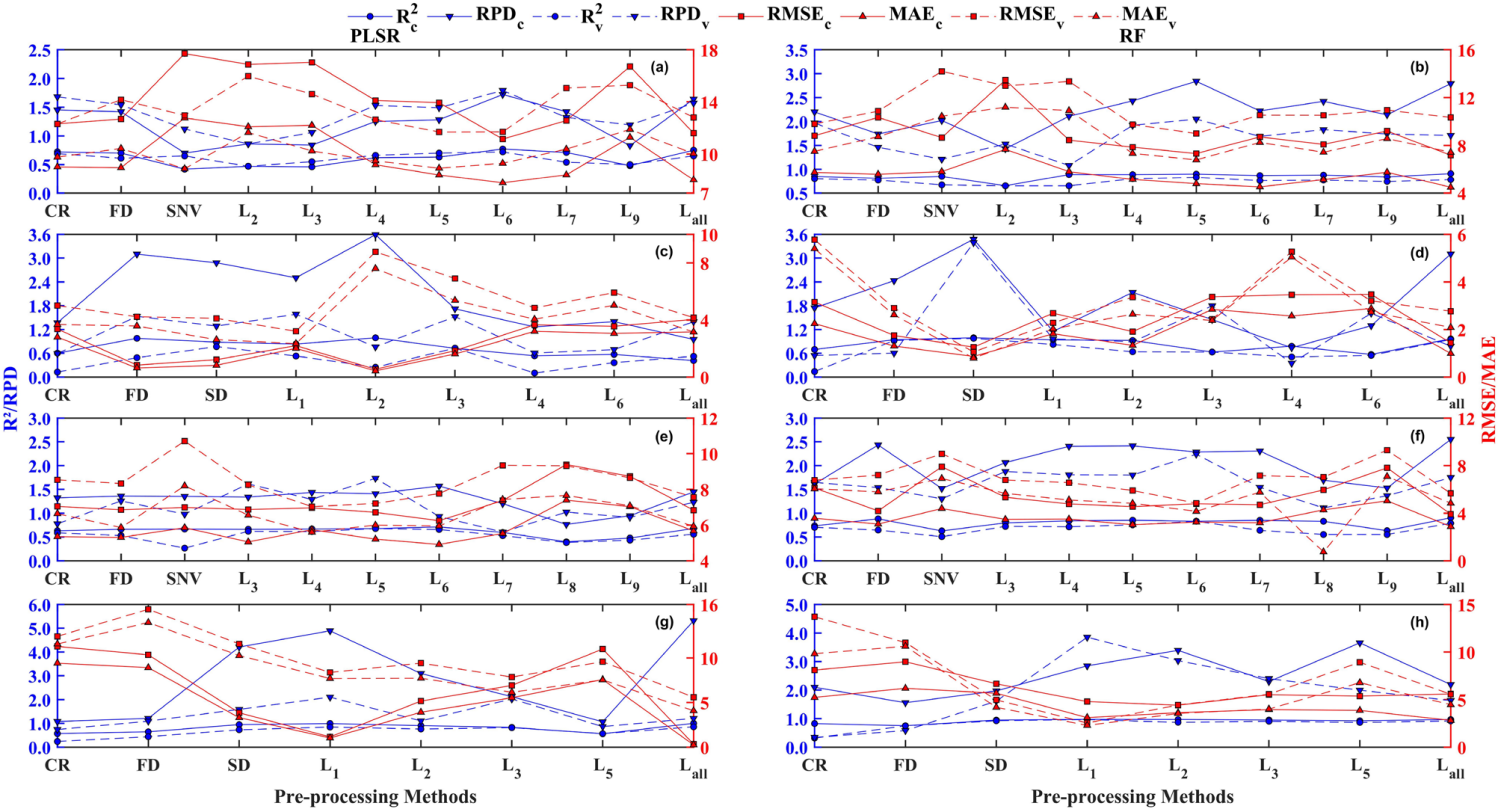
Comparing the performance of calibrating Zn concentration corrected by natural logarithmic transformation based on different spectral reflectance transformation methods using RF and PLSR at four conditions concerning the entire group, clean group, low pollution group, and moderate pollution group. (**a**, **b**): entire samples group, (**c**, **d**): clean group, (**e**, **f**): low pollution group, and (**g**, **h**): moderate pollution group.

**Figure 7:**
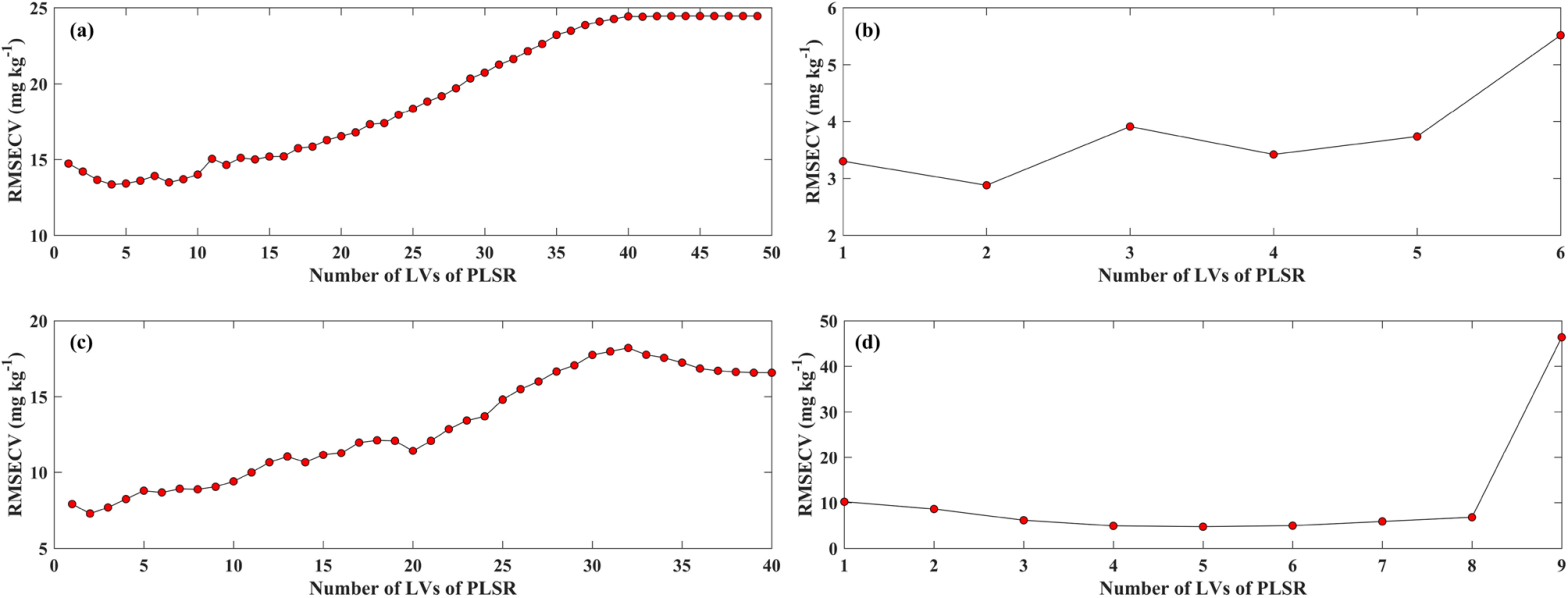
The scatter plots for determining the lowest RMSECV and the corresponding number of LVs. The number of LVs with the lowest RMSECV was used to fit the optimal PLSR in estimating Zn concentrations at four conditions concerning the (**a**) entire group based on L_6_, (**b**) clean group based on L_3_, (**c**) low pollution group based on L_5_, and (**d**) moderate pollution group based on L_1_.

Some reflectance spectra transformation methods were not illustrated in Fig. 6 because the characteristic bands can hardly be retrieved through the above methods. For the entire group, the result of RF based on L_5_ with relatively higher R^2^ (R^2^ = 0.83), RPD (RPD = 2.05), and lower MAE (MAE = 6.79 mg kg^−1^), as well as RMSE (RMSE = 9.00 mg kg^−1^), outperformed PLSR using L_6_ with lower R^2^ (R^2^ = 0.72), RPD (RPD = 1.79), and higher MAE (MAE = 9.30 mg kg^−1^), RMSE (RMSE = 11.71 mg kg^−1^). RMSECV reached the minimum value when LVs were 4 (Fig. 7a). Besides, the best performance was found as the RF (ntree = 4, mtry = 3) combined with L_5_ was trained for estimating Zn concentration corrected by natural logarithmic transformation (Fig. 6a,b). For the clean group, the result of RF-based on SD with relatively higher R^2^ (R^2^ = 0.97), RPD (RPD = 3.39), and lower MAE (MAE = 0.79 mg kg^−1^), as well as RMSE (RMSE = 1.26 mg kg^−1^), was better than PLSR using L_3_ with lower R^2^ (R^2^ = 0.73), RPD (RPD = 1.52) and higher MAE (MAE = 5.37 mg kg^−1^), RMSE (RMSE = 6.90 mg kg^−1^). RMSECV reached its minimum value when LVs was 2 (Fig. 7b). Additionally, the optimum robustness has appeared when the RF (ntree = 96, mtry = 3) combined with SD was fitted (Fig. 6c,d). For the low pollution group, the outcome of RF-based on L_6_ with relatively higher R^2^ (R^2^ = 0.83), RPD (RPD = 2.24), and lower MAE (MAE = 4.15 mg kg^−1^), as well as RMSE (RMSE = 4.84 mg kg^−1^), surpassed PLSR using L_5_ with lower R^2^ (R^2^ = 0.68), RPD (RPD = 1.73), and higher MAE (MAE = 5.99 mg kg^−1^), RMSE (RMSE = 7.21 mg kg^−1^). Two LVs existed in the PLSR calibration (Fig. 7c). Also, the optimal method for estimating Zn concentration corrected by natural logarithmic transformation was RF (ntree = 3, mtry = 13) combined with L_6_ (Fig. 6e,f). For the moderate pollution group, the result of RF-based on L_1_ with relatively higher R^2^ (R^2^ = 0.96), RPD (RPD = 3.85), and lower MAE (MAE = 2.54 mg kg^−1^), as well as RMSE (RMSE = 4.79 mg kg^−1^), outperformed PLSR using L_1_ with lower R^2^ (R^2^ = 0.84), RPD (RPD = 2.10), and higher MAE (MAE = 7.69 mg kg^−1^), RMSE (RMSE = 8.37 mg kg^−1^). Four LVs were determined for PLSR calibration (Fig. 7d). Also, the best method for examing Zn concentration corrected by natural logarithmic transformation was RF (ntree = 31, mtry = 9) combined with L_1_ (Fig. 6g,h).

**Figure 8:**
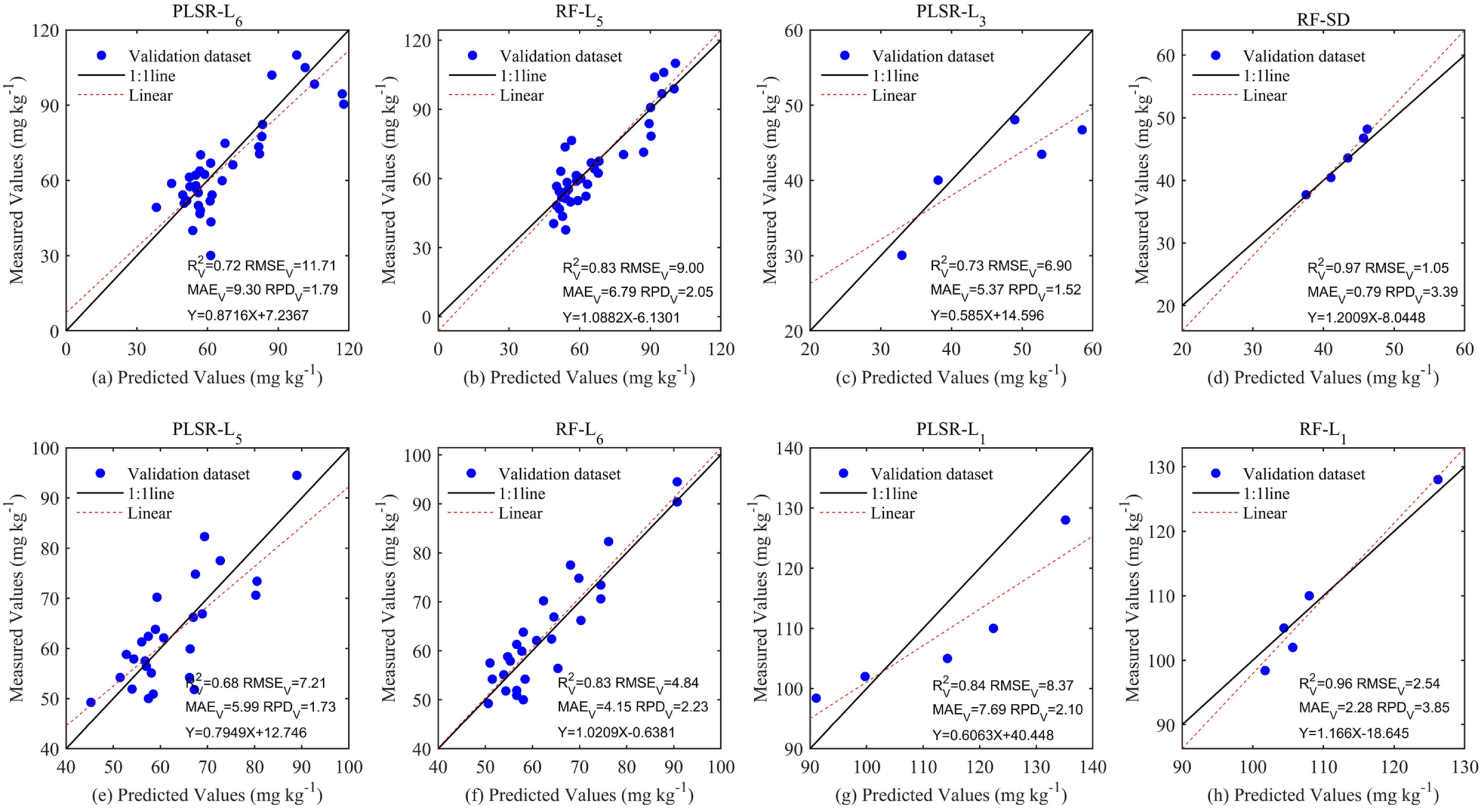
Scatter plots for predicting Zn concentration based on the optimal calibration models at four conditions concerning the entire group (N = 111), clean group (N = 14), low pollution group (N = 81), and moderate pollution group (N = 16). (**a**): entire samples group using PLSR model combined with L_6_, (**b**): entire samples group using RF model combined with L_5_ (**c**): clean group using PLSR model combined with L_3_, (**d**): clean group using RF model combined with SD, (**e**): low pollution group using PLSR model combined with L_5_, (**f**): low pollution group using RF model combined with L_6_, (**g**): moderate pollution group using PLSR model combined with L_1_, and (**f**): moderate pollution group using RF model combined with L_1_.

Overall, RF was better than PLSR no matter which group was trained in estimating Zn concentration corrected by natural logarithmic transformation. Moreover, the CWT method outperformed the others in the majority of situations (Fig. 6). So, the L_6_ combine with PLSR (PLSR-L_6_), the L_5_ combine with RF (RF-L_5_), the L_3_ combine with PLSR (PLSR-L_3_), the SD combine with RF (RF-SD), the L_5_ combine with RF (PLSR-L_5_), the L_6_ combine with RF (RF-L_6_), the L_1_ combine with PLSR (PLSR-L_1_), and the L_1_ combine with RF (RF-L_1_) were chosen to map the scatter plot for validating the robustness of each method in examing Zn concentration corrected by natural logarithmic transformation (Fig. 8)^93^. Obviously, the best performance was detected in the clean group with the highest validation R^2^ (R^2^ = 0.97), RPD (RPD = 3.39), and relatively lower MAE (MAE = 0.79 mg kg^−1^), RMSE (RMSE = 1.05 mg kg^−1^) (Fig. 8d). Other results concerning calibration and validation with relatively lower R^2^, RPD, as well as higher MAE and RMSE were presented in Fig. 8a,b,c,e–g.

## Discussion

### The possible reasons for Zn represented a skewness distribution

The mean Zn concentration (67.98 mg kg^−1^) of the study area was larger than the background value of the Inner Mongolia Autonomous Region (48.6 mg kg^−1^), which may be led by the coal resources development (Table 1). Moreover, the CV (0.33) indicated that the Zn distribution may be influenced by human activities (Table1). On the one hand, the Zn distribution revealed obvious spatial heterogeneity that the concentration of soil samples close to the mining areas was relatively higher than the distant samples (Fig. 1). The mean content for the soil samples near and off the mining areas was 72.27 mg kg^−1^, and 56.63 mg kg^−1^, respectively. On the other hand, the Zn content may be affected by the distribution of roads in the mining areas because the coal was transported via trucks to the outside and the previous studies proved that one of the major sources for Zn is the worn tires^66^. So, the skewed distributions of Zn were observed in the current study area (Fig. 3).

### The spectral reflectance of the soil samples in the present study area may be affected by the Zn content

Figure S2SG was chosen for describing the character of the soil sample spectral reflectance because the original spectrum of the soil sample was only smoothed but did not change the basic spectral features by the SG spectral transformation method. Furthermore, Fig. S2SG showed that the spectral reflectance varied with the spectral wavelength and was separated by the content of the Zn, especially during 580–1850 nm. The spectral reflectance was decreased with the Zn content increasing, that is, the higher content of Zn exhibited, the lower the spectral reflectance represented. The correspondence results were reported by some published studies. For example, the soil samples from varying metals groups revealed similar spectral features but with variable spectral intensities for the electromagnetic energy was absorbed during some specific wavelength, so the spectral curve changed with the metals content. Overall, the spectral reflectance tended to decrease with the increase in the metals content^45^. Chakraborty et al.^91^ and Douglas et al.^94^ concluded similar characteristics and revealed that polluted soil samples represented a stronger spectral absorbance than the unpolluted soil samples, particularly in the spectral range of 700–2500 nm which was in line with our results.

### The number of characteristic bands varies with the condition of measuring spectral reflectance, spectral reflectance transformation methods

There were no characteristic bands derived from in-situ and lab-based unprocessed situations in the current study. Contrarily, plenty of characteristic bands were obtained under the lab-based processed condition that implied some possible factors may absorb spectral energy. Soil reflectance spectrum is a cumulative reflection of the physical and chemical properties of soil. Previous studies confirmed that soil properties concerning soil moisture, parent material, organic matter, iron oxides, particle size, mineralogy, and soil structure exhibited significant influence on soil reflectance^21,95^. For the lab-based unprocessed and in-situ situation, soil properties such as particle size and moisture content may generate an influence on soil spectral measurement^70^. Besides, the soil surface condition can also affect soil reflectance^23^. Therefore, probable factors that limit the number of characteristic bands under lab-based unprocessed and in-situ situations are potential environmental factors. Whereas, for the lab-based processed situation, the soil samples were processed via air-dried, sieved, and grounded into fine particles before spectral surveying to exclude the effect of soil structure, soil moisture, and particle size on spectral characteristics of soil samples.

Clearly, for the lab-based processed situation, the number of characteristic bands based on CWT is obviously larger than other spectral reflectance transformation methods no matter which concentration group and the entire samples were selected for training (Fig. S9). The probable reason why CWT can effectively extract characteristic bands is that CWT offers variable time–frequency resolution that can efficiently and precisely capture time-series information. CWT, with variable size windows, is a windowing technique. Smaller and larger time intervals can be used for analyzing the high and low frequencies through CWT. Both the time domain and the frequency domain were widely used to capture the local patterns of the signal. So, the spectral features could be extracted more effectively via CWT^50^.

### The accuracy for estimating Zn concentration depends on the pretreatment for the soil samples, spectral reflectance transformation methods, and the calibration models

The accuracy of the calibration for Zn concentration was discussed in the present study. First, the results demonstrated that the spectral transformation method may influence accuracy. The CWT was the best one than others including CR, FD, SD, SG, ABS, MSC, and SNV in estimating Zn content. Some similar outcomes have also been published in previous studies^95,96^. The possible reason for CWT can improve the accuracy of model estimation was similar to characteristic bands selection discussed in Sect. 4.3. Although the baseline drift caused by the differences in grinding and optical setups can be minimized via MSC and SNV^49^, the benefit is less for the baseline does not change very much. The derivatives can be used in the condition that the low noise level is ensured^97^. In this study, the accuracy of FD and SD was relatively weak because severe noise existing in the original spectrum influenced the valuable information to be extracted^45^. Second, the outcomes revealed that the calibration methods may also affect accuracy. The RF method exhibited significantly higher accuracy than the PLSR in estimating Zn concentration using Vis–NIR spectral data because the relationship between Zn content and spectral reflectance is not linear but non-linear. Though the PLSR can efficiently deal with the collinearity issue, the capability for solving non-linear relationships is relatively weak^53,98^. The RF model with insensitivity to outliers and excellent generalization ability outperformed PLSR in calibrating Zn concentration. Additionally, the model performance may be influenced by a wide range of target properties^99^. The optimal model for estimating Zn concentration was the RF model due to the relatively wide range of Zn variations in this study (CV = 0.33). On the contrary, the PLSR method had relatively poor applicability when the sample range is very large. Third, the accuracy was also affected by the pretreatment conditions of soil samples^45^. For the in-situ and lab-based unprocessed situations, there were no obvious characteristic bands because soil properties revealed significant influence on soil spectral reflectance resulting in poor capability for calibrating Zn concentrations.

The soil samples were divided into three groups according to the Zn content including clean, low pollution, and moderate pollution groups to test the calibration results that were influenced by the concentration of Zn or not. The accuracy was promoted with the Zn concentration increasing when the CWT with L_3_ scale was selected to fit the RF model (Fig. 6d,f,h). Whereas, the accuracy represented decreasing trend with the Zn concentration increasing no matter which calibration methods we used when the FD was chosen to run the model (Fig. 6c-h). So, the accuracy of calibration may not be affected by the content of Zn.

The relationship between the accuracy of the calibration model and the number of characteristic bands was not significant. However, the accuracy may be improved through retrieving characteristic bands with highly R^2^ that played an important role in calibrating Zn content (Figs. 4, 5, 6). The characteristic bands with the highest R^2^ appeared in the results of the moderate pollution group (R^2^ = 0.71), followed by the clean group (R^2^ = 0.69), the entire samples group (R^2^ = 0.61), and the low pollution group (R^2^ = 0.59). The R^2^ for the model validation were 0.96, 0.83, 0.97, and 0.83 for the moderate pollution group, the clean group, the entire samples group, and the low pollution group, respectively.

Overall, we speculated that the accuracy of calibration may be influenced by spectral reflectance transformation methods, calibration methods, the condition for measuring soil spectrum, and the characteristic bands with highly R^2^ that were used to describe the relationship between the Zn content and the reflectance spectra after transforming. However, the content of Zn, the number of the samples, and the number of the characteristic bands excluding the results obtained from RF represented a relatively weak effect in the accuracy of estimating Zn content.

### The possible reasons for the important bands in estimating Zn concentrations based on PLSR and RF models using Vis–NIR spectra

**Figure 9:**
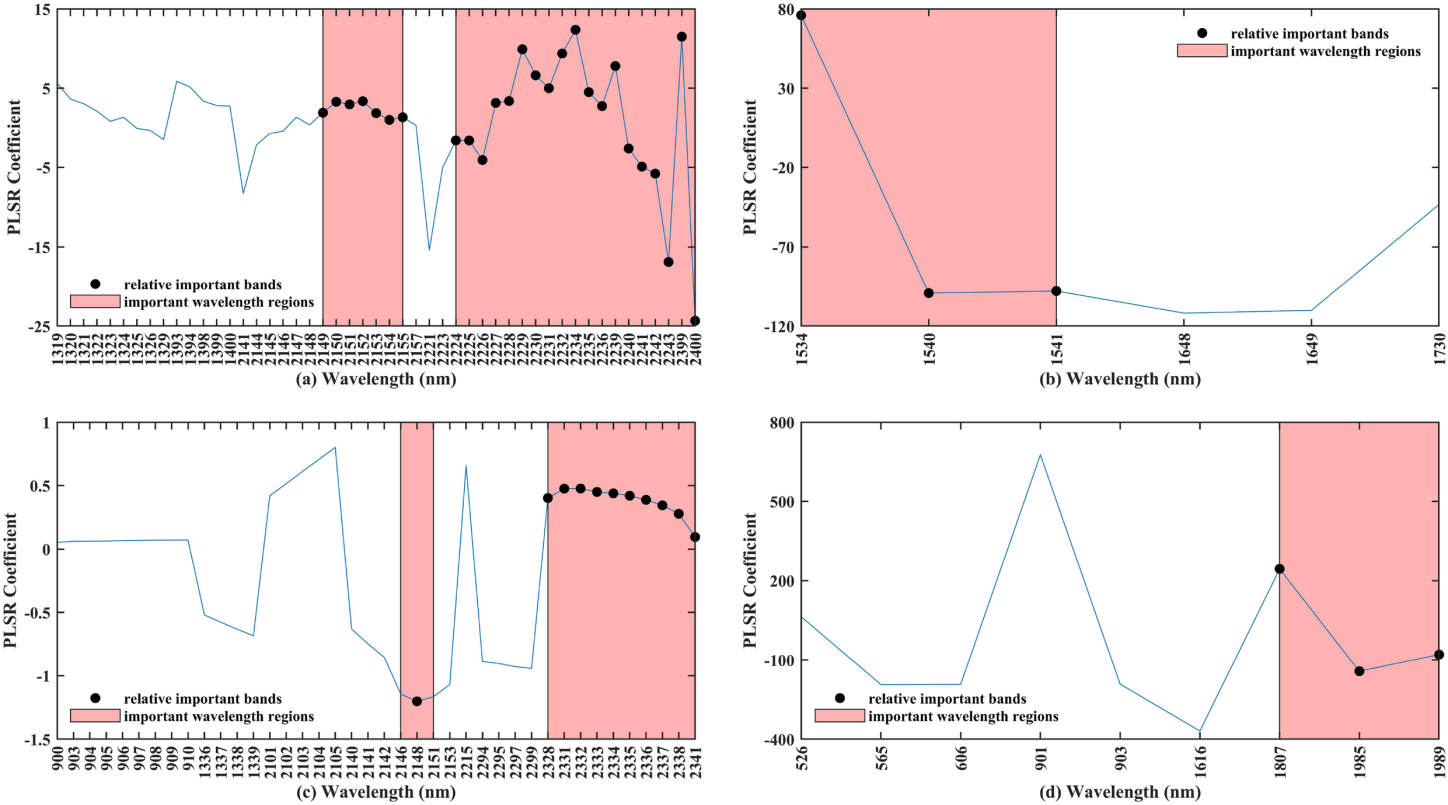
The distribution plots of important wavelength regions, relative important bands, and the coefficients of optimal PLSR for inferring Zn concentration at four conditions concerning the (**a**) entire group based on L_6_, (**b**) clean group based on L_3_, (**c**) low pollution group based on L_5_, and (**d**) moderate pollution group based on L_1_.

**Figure 10:**
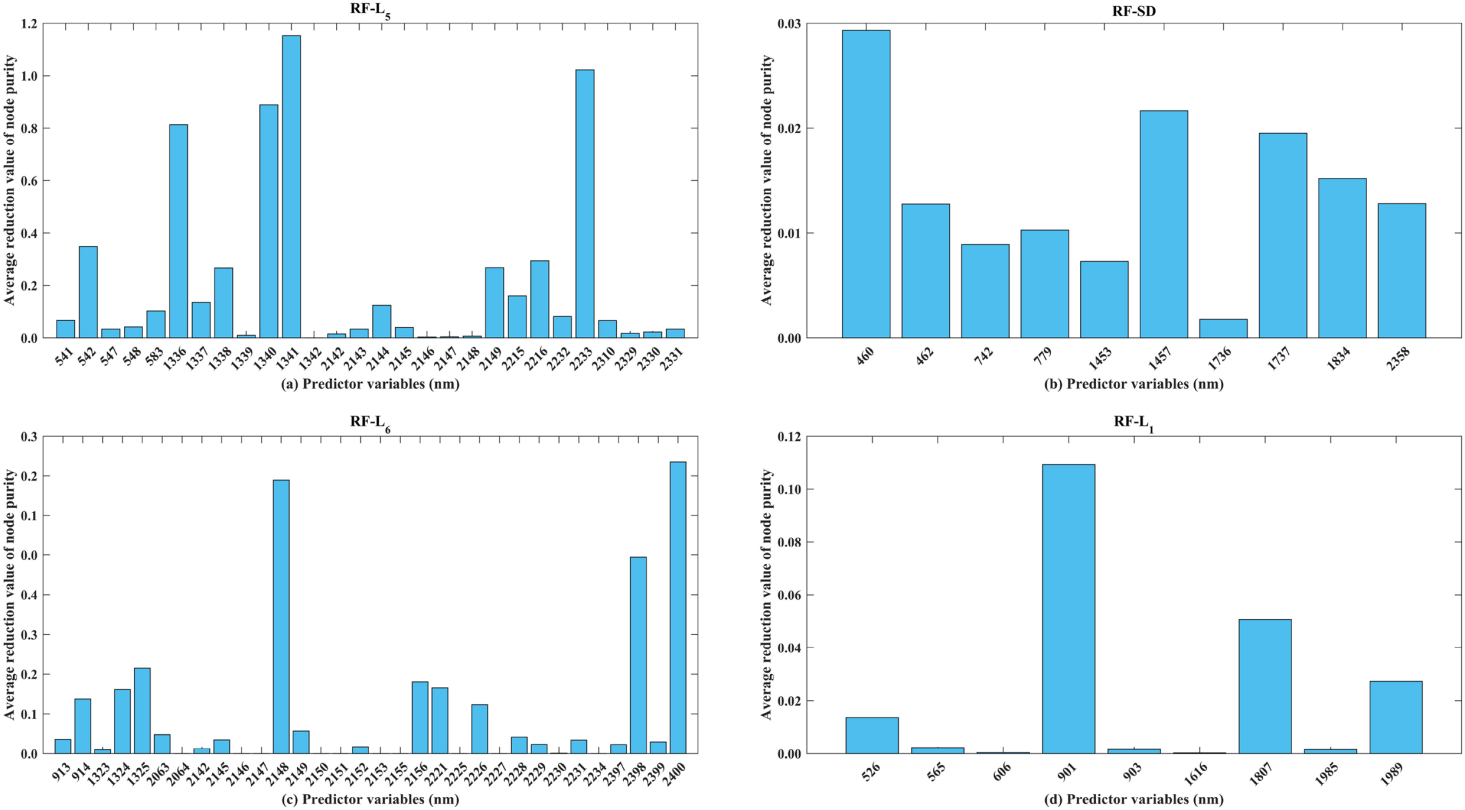
The histogram of importance bands for RF in Zn concentration estimation at four conditions concerning the (**a**) entire group, (**b**) clean group, (**c**) low pollution group, and (**d**) moderate pollution group. Note: The more important the variable is, the larger average reduction value of node purity values is.

Several important bands for the optimal PLSR including 1534, 1540, 1541, 1807, 1985, 1989, 2148–2155, 2224–2243, 2328, 2331–2338, 2341, 2399, and 2400 nm were proved their importance for Zn estimation (Fig. 9a-d). For the optimum RF model, 460, 901, 1336, 1340, 1341, 1457, 1737, 1807, 1834, 1989, 2148, 2233, 2398, and 2400 nm were identified as the relative important bands (Fig. 10a-d). To our knowledge, clay minerals demonstrated the strong adsorption of Zn around 1400, 1900, and 2200 nm^87^. Specifically, the spectral reflectance features of clay minerals demonstrated kaolinite spectrum had an obvious feature owing to a hydroxyl absorption wavelength with aluminum coordination at around 2200 nm. Two significant absorption regions about 1400 nm and 1900 nm were detected for vermiculite had interlayer moisture. Furthermore, a previous study proved that spectral bands associated with organic matter and clay minerals can be used for inferring Zn concentration with relatively high estimation accuracy^87^. Meanwhile, an experiment designed for exploring the relationship between heavy metals and soil constituents confirmed Zn was easily absorbed on soil mineral constituents, especially montmorillonite and vermiculite under the competitive environment^100^. Moreover, published researches confirmed that soil organic matter and goethite also represented obvious adsorption of Zn at 400–800 nm and 420 and 950 nm,respectively^45,101^.

### Research limitations and future research plans

There are some problems that need to be further addressed. First, the current study was a local research, not a global finding, the methods used in the present study may exist drawbacks due to the universal application. Second, though the calibration models for inferring heavy metals concentrations were obtained, the models of this study only can be used to estimate the heavy metals concentrations at sampling sites. Obtaining continuous heavy metals concentrations distribution is strongly desired. So, exploring calibration models using space-borne and airborne sensing combined with the ground level models based on in-situ sampling sites is still a big challenge. Third, some related soil properties concerning the soil texture, pH, salt, organic matter, clay minerals, and the presence of other heavy metals, combined with natural environmental factors, such as soil parent material and soil formation conditions were ignored in the current study. So, the accuracy of this study needs to be improved. Fourth, the present study only constructed the calibration model for the Zn element. Other toxic metals need to be studied in the future. Fifth, the spectral response of heavy metals was very weak for heavy metals in soils are truly a minute component comparing with water, organic matter, and clay minerals. It is impossible to detect the spectral signal of some metals unless their content exceeds 4.0 mg g^−1^. So, we plan to deep dive into this orientation as follows. First, the space-borne and airborne sensing products will be implemented to retrieve the metals distribution at large scales. In the future, we plan to (1) utilize a direct standardization algorithm for establishing a transfer model of soil spectra between laboratory obtained and GaoFen-5 image obtained to inverse the soil heavy metals concentrations, and (2) to obtain the continuous distribution map of the soil heavy metals concentrations based on the optimal estimation model determined by the RF, extreme learning machine (ELM), support vector machine (SVM), and back-propagation neural network (BPNN) algorithms for the study area. Second, the related soil properties will be considered in the next research for improving accuracy. Third, we plan to continually develop methods in improving the accuracy of the calibration model using novel deep learning methods. In general, the mechanism of metals concentrations is very complicated, and the metals concentrations may be affected by both anthropic and natural factors. Moreover, the relations of metals concentrations with soil properties are still not very clear. Although pure metals were hard to sorb vis–NIR and mid-IR radiation, the correlated relationship between heavy metals concentrations and organic matter as well as clay minerals can be used for inversing heavy metals indrectly. Fourth, Ni, Cu, and Pb will be considered in the future due to the serious toxicity. Meanwhile, the Cr element will be also chosen as the objective because the Cr element was considered as common heavy metals pollutants in an open pit coal mine. Overall, it is still a huge challenge to conduct a multidisciplinary study in estimating metals concentrations.

## Conclusion

The present study revealed that Vis–NIR spectroscopy can be used to calibrate Zn concentration in topsoils of open cast coal mining areas. Overall, the spectral reflectance tended to decrease with the increase of the Zn content during 580^−1^850 nm based on SG smoothing. CWT could retrieve more detailed spectral characteristics than other methods mainly because CWT can offer variable time–frequency resolution that can efficiently and precisely capture time-series information. The RF combined with CWT demonstrated the optimal accuracy than other methods in the current study (calibration: R^2^ = 0.99, RPD = 3.47, RMSE = 1.26 mg kg^−1^, MAE = 0.86 mg kg^−1^; validation: R^2^ = 0.97, RPD = 3.39, RMSE = 1.05 mg kg^−1^, MAE = 0.79 mg kg^−1^). The accuracy of estimating Zn content may be hardly affected by the content of Zn, the number of the samples, and the number of the characteristic bands excluding the results obtained from RF. This study will help to develop an effective technique to speedily detect metals concentration in possible contaminated areas such as coal mines and metallic mineral deposit areas.

## Supplementary Information

Supplementary Information.
