# Supplementary material for: Retrieving zinc concentrations in topsoil with reflectance spectroscopy at Opencast Coal Mine sites
Source: Sci Rep. 2021 Oct 7;11:19909. doi: 10.1038/s41598-021-99106-1 (PMC8497582; doi:10.1038/s41598-021-99106-1)
Supplement: Supplementary file 1 — Supplementary Information. [file 41598_2021_99106_MOESM1_ESM.docx]

**Retrieving Zinc concentration in topsoil with reflectance spectroscopy at Opencast Coal Mine sites**

Bin Guo ^1, *, a^, Bo Zhang ^1, a^, Yi Su ^1^, Dingming Zhang ^1^, Yan Wang ^1^, Yi Bian ^1^, Liang Suo ^1^, Xianan Guo ^1^, Haorui Bai^1^

^1^ College of Geomatics, Xi’an University of Science and Technology, Xi’an, China

^*^ Corresponding author: Bin Guo, E-Mail: guobin12@xust.edu.cn

^a^ These authors contributed equally to this work.

Supporting Information

Contents of this file

Figure S1-S9

Tables S1-S2

Figure S1. Three sorts of soil spectra of the present study area. (a) Lab-based processed spectra, (b) Lab-based processed spectra after removing noisy regions (350-399 nm, 2400-2500 nm), (c) Lab-based unprocessed spectra, (d) Lab-based unprocessed spectra after removing noisy regions (350-399 nm, 2400-2500 nm), (e) In-situ spectra, and (f) In-situ spectra removing spectral noise aroused by atmospheric water vapor.


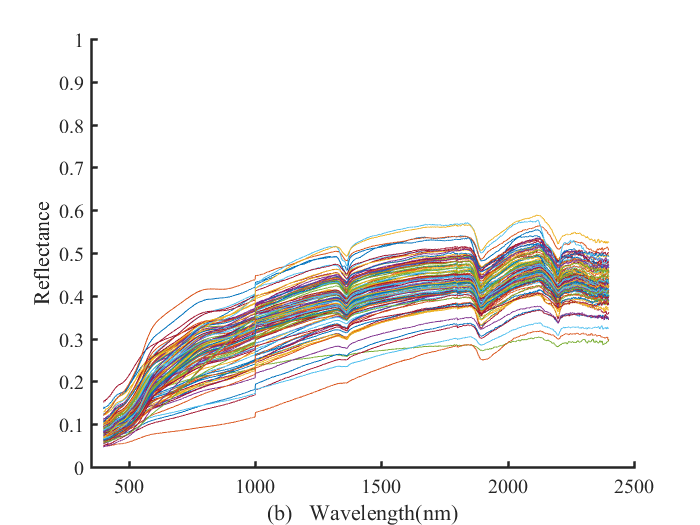

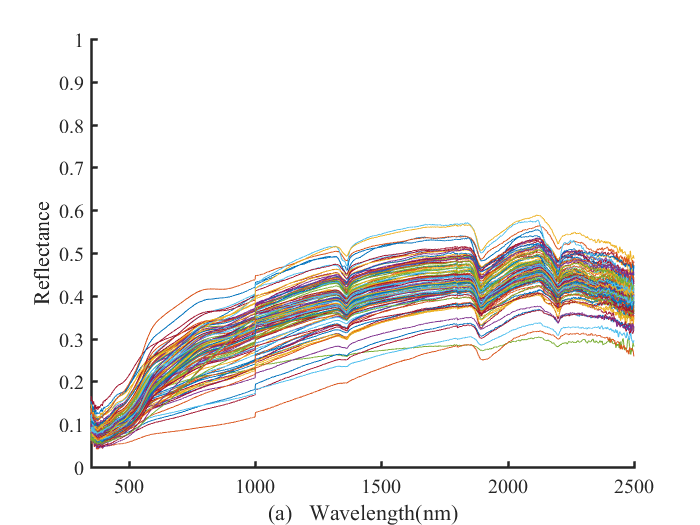

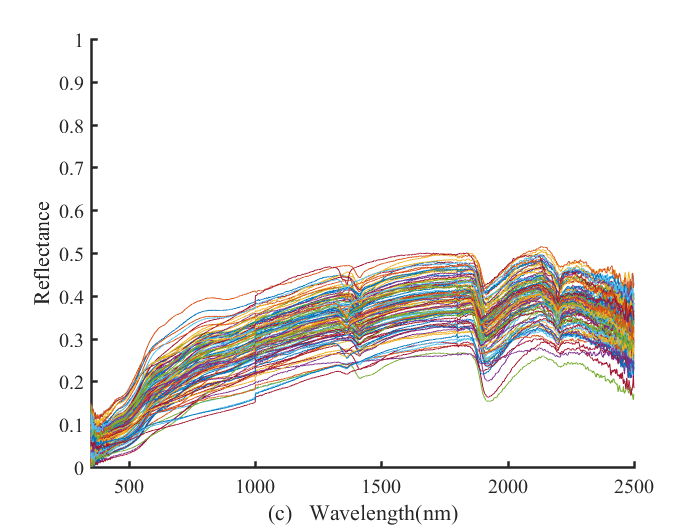

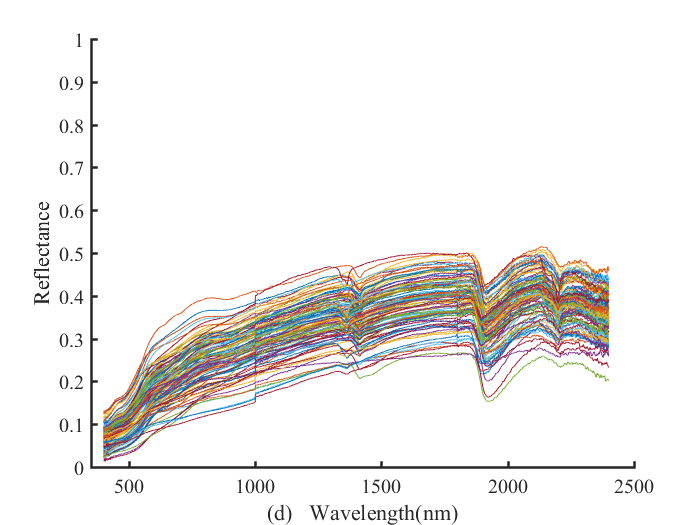

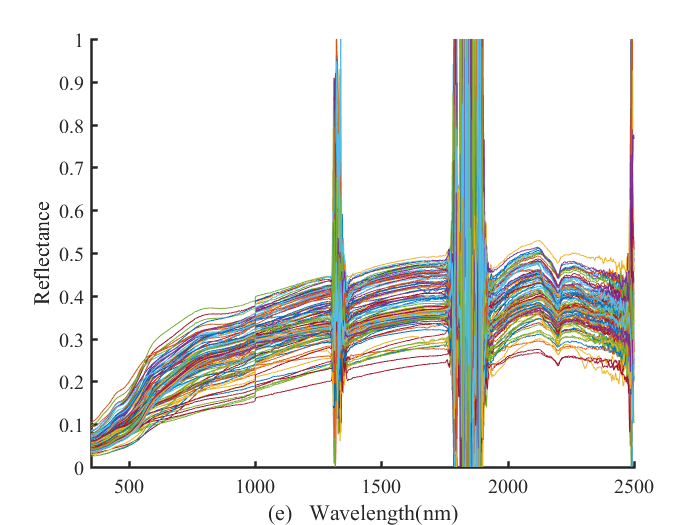

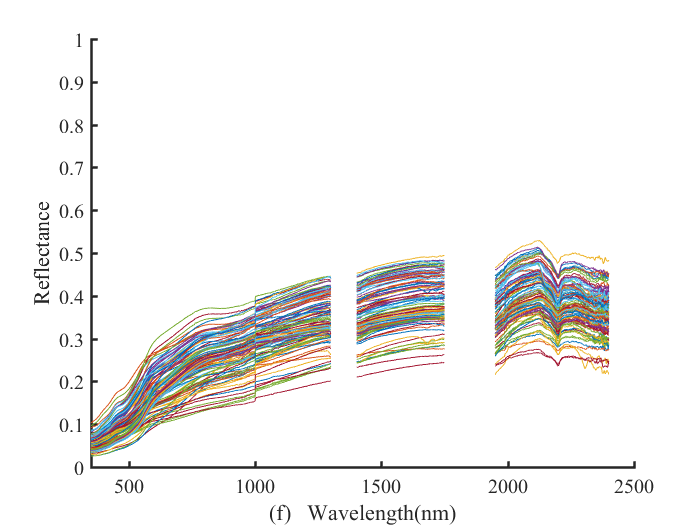


**
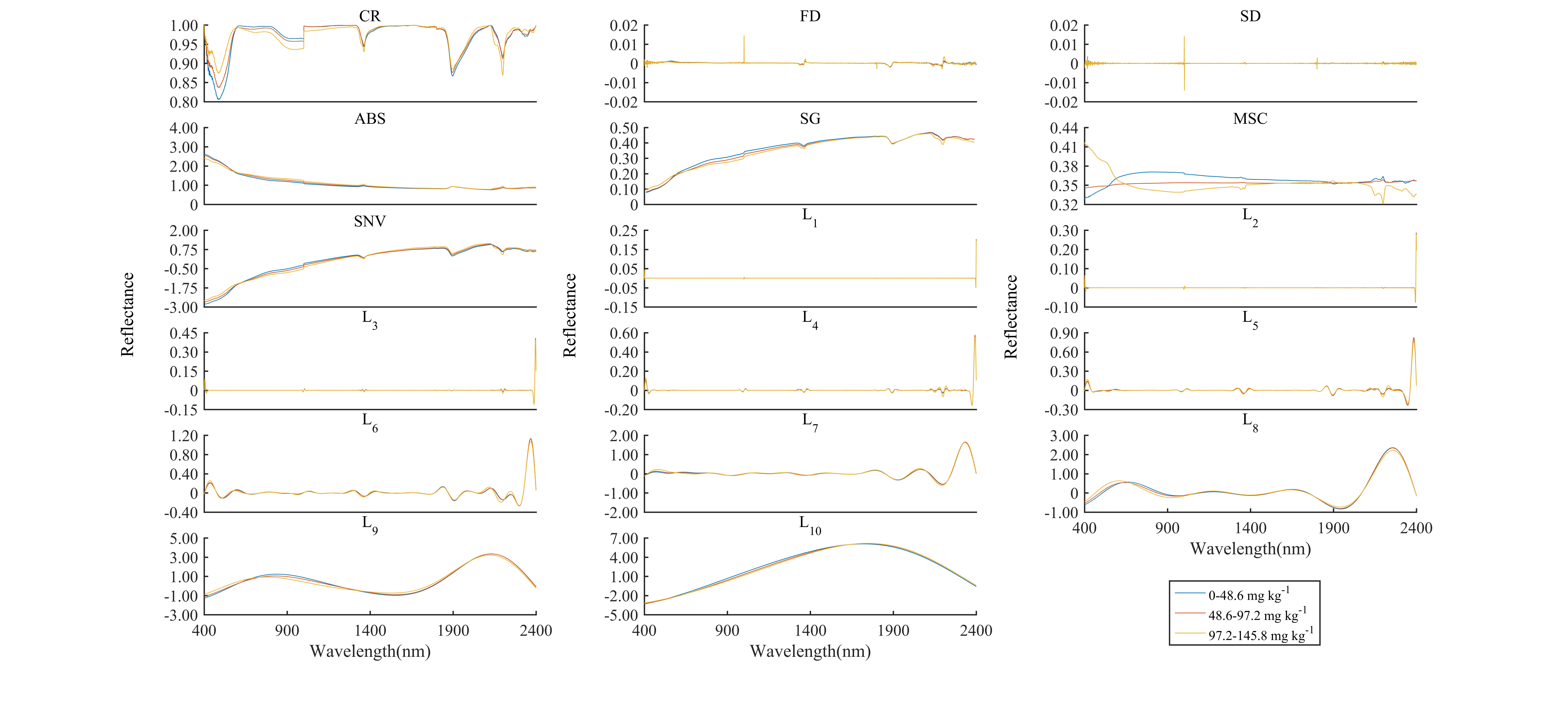
**

Figure S2. Comparison of the soil spectral reflectance curves under lab-based processed for three sorts of Zn concentration groups based on the background value (BV) of the Inner Mongolia Autonomous Region using the contamination factor method, including clean (Zn ≤ 48.6 mg kg^-1^), low pollution (48.6 ≤ Zn ≤ 97.2 mg kg^-1^), and moderate pollution (97.2 ≤ Zn ≤ 145.8 mg kg^-1^), respectively using eight spectral preprocessing methods.

(Note: L_1_-L_10_ denoted the reconstructed spectral reflectance curves using CWT at decomposition scales of 1-10.)


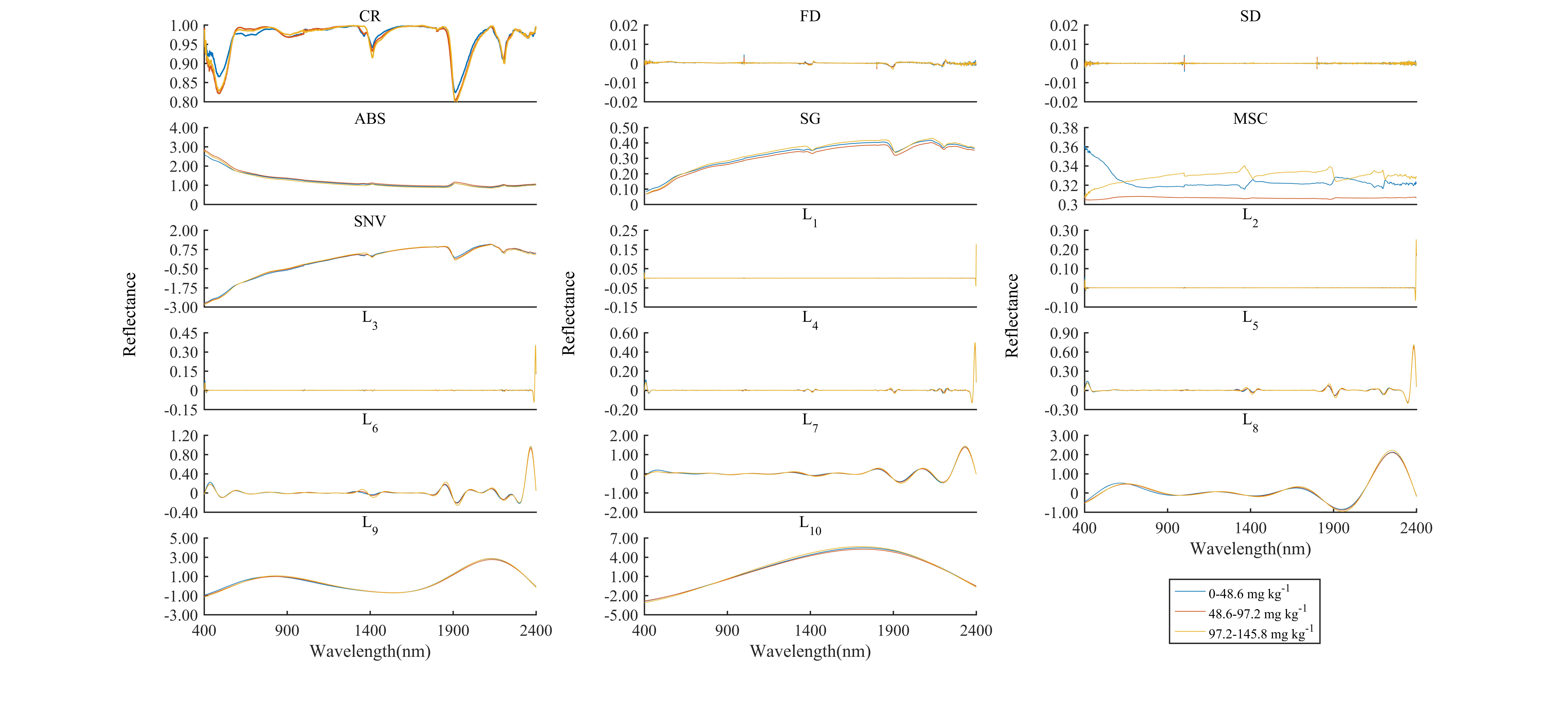
Figure S3. Comparison of the soil spectral reflectance curves under lab-based unprocessed for three sorts of Zn concentration groups based on the background value (BV) of the Inner Mongolia Autonomous Region using the contamination factor method, including clean (Zn ≤ 48.6 mg kg^-1^), low pollution (48.6 ≤ Zn ≤ 97.2 mg kg^-1^), and moderate pollution (97.2 ≤ Zn ≤ 145.8 mg kg^-1^), respectively using eight spectral preprocessing methods.

(Note: L_1_-L_10_ denoted the reconstructed spectral reflectance curves using CWT at decomposition scales of 1-10.)


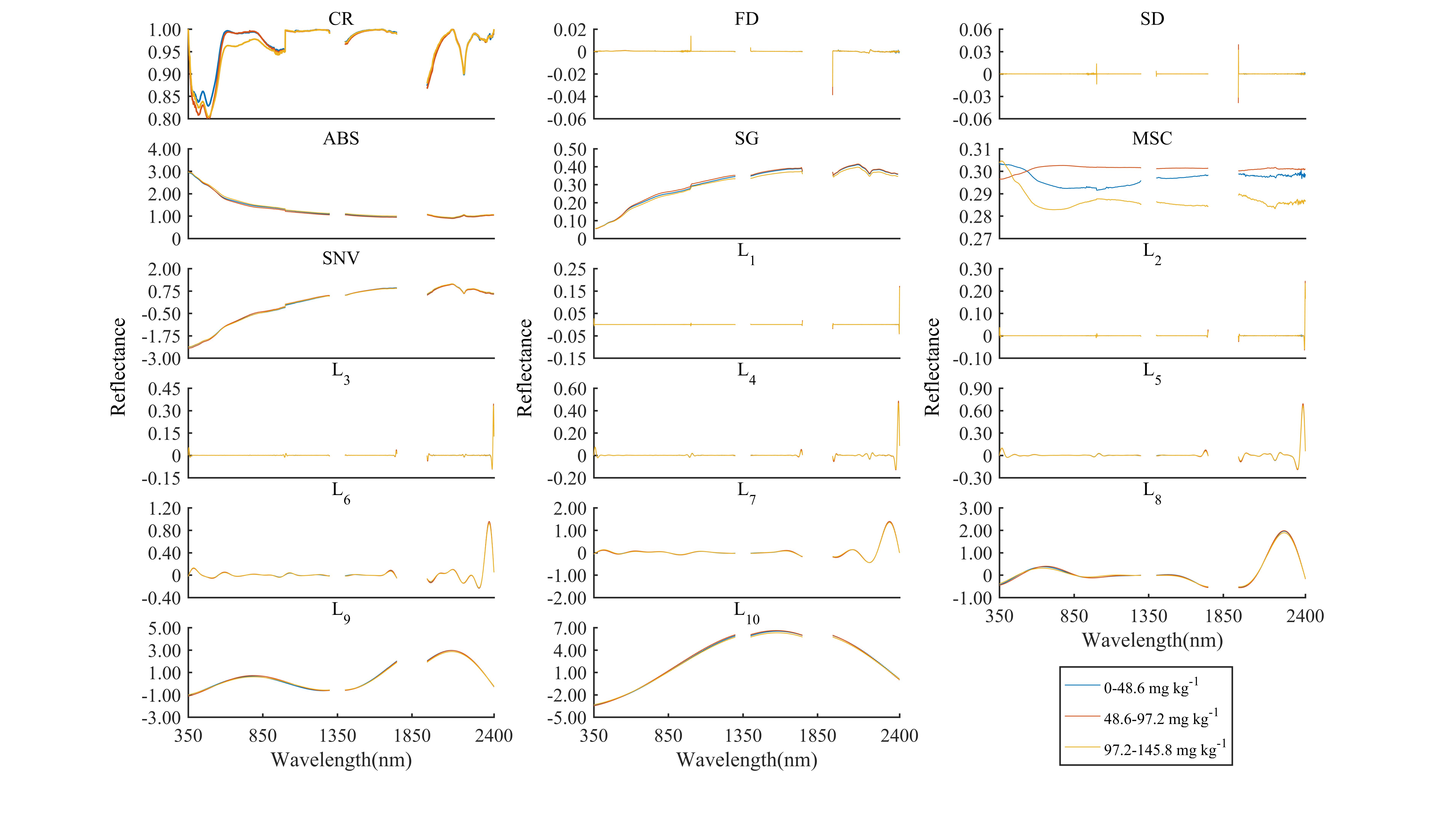


Figure S4. Comparison of the soil spectral reflectance curves under in-situ for three sorts of Zn concentration groups based on the background value (BV) of the Inner Mongolia Autonomous Region using the contamination factor method, including clean (Zn ≤ 48.6 mg kg^-1^), low pollution (48.6 ≤ Zn ≤ 97.2 mg kg^-1^), and moderate pollution (97.2 ≤ Zn ≤ 145.8 mg kg^-1^), respectively using eight spectral preprocessing methods.

(Note: L_1_-L_10_ denoted the reconstructed spectral reflectance curves using CWT at decomposition scales of 1-10.)


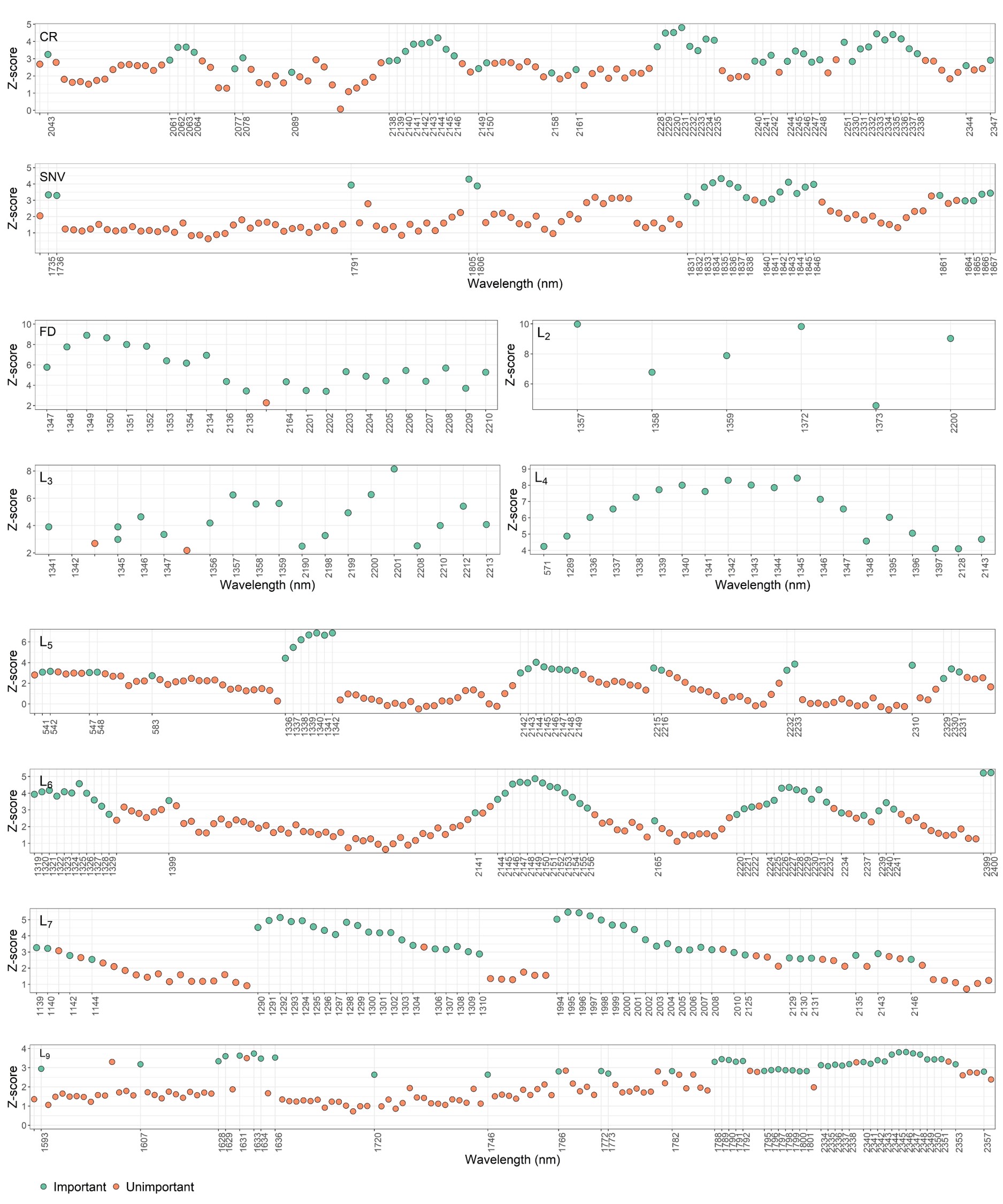


Figure S5. The position of optimal characteristics bands based on the Boruta algorithm using Z-score for the lab-based processed situation.





Figure S6. The position of optimal characteristics bands based on the Boruta algorithm using Z-scores for clean group situation.

Figure S7. The position of optimal characteristics bands based on the Boruta algorithm using Z-scores for low pollution group situation.










Figure S8. The position of optimal characteristics bands based on the Boruta algorithm using Z-scores for moderate pollution group situation.





Figure S9. The number of characteristic bands under lab-based processed situation for four sorts of Zn concentration groups including (a) clean (N=14), (b) low pollution (N=81), (c) moderate pollution (N=16), and (d) the entire samples (N=111), respectively

| Sorts | Wavelength (nm) |
| --- | --- |
| Lab-based processed | 1347-1354; 1699-1867; 2041–2096; 2132-2174; 2196-2210; 2218-2251; 2330-2347 |
| Clean group | 460-462; 481; 742; 744; 779; 955; 960; 1073; 1206; 1248; 1249; 1297-1299; 1342; 1389; 1453; 1457; 1458; 1543; 1602; 1644; 1736; 1737; 1758; 1775; 1788; 1834; 1915; 1922; 2030; 2031; 2035; 2251; 2264; 2320; 2358 |
| Low pollution group | 1348-1352; 1833-1835; 1839-1863; 2050-2065; 2075-2090; 2133-2166; 2201-2210; 2222-2248; 2330-2346 |
| Modermate plolltion group | 414; 471; 475; 526; 543; 545; 812; 840; 846; 848; 849; 900; 939; 1083; 1084; 1192; 1548; 1549; 1570; 1571; 1605; 1615; 1616; 1653; 1809; 1983; 1987; 2025; 2097; 2098; 2220; 2251-2253; 2282 |

Table S1. The position of rough characteristic bands determined by CR, FD, SD, SG, ABS, MSC, and SNV methods

Note: soil samples were classified into three types based on the background value (BV) of the Inner Mongolia Autonomous Region using the contamination factor method, including clean (Zn ≤ 48.6 mg kg^-1^), low pollution (48.6 ≤ Zn ≤ 97.2 mg kg^-1^), and moderate pollution (97.2 ≤ Zn ≤ 145.8 mg kg^-1^), respectively. The rough characteristic bands were not observed in lab-based unprocessed and in-situ situations.

| Sorts | Wavelength (nm) |
| --- | --- |
| Lab-based processed | 526-554; 571-573; 584-598; 1139-1158; 1288-1310; 1296; 1318-1330; 1336-1348; 1352-1359; 1372; 1373; 1375-1385; 1393-1402; 1417-1419; 1444-1449; 1550-1802; 1994-2010; 2058-2093; 2097-2253; 2262-2310; 2326-2373; 2399; 2400 |
| Clean group | 480; 742; 744; 774; 778; 1242; 1259; 1343; 1456; 1501; 1541; 1546; 1736; 1921; 2032; 2158; 2319 |
| Low pollution group | 400-413; 434-468; 524-549; 568-586; 877-879; 898-917; 934-1017; 1323-1325; 1336-1347; 1341-1347; 1357-1359; 1376-1380; 1488-1742; 2062-2066; 2097-2099; 2100-2122; 2136-2163; 2215-2240; 2242-2251; 2270-2305; 2323-2348; 2397-2400 |
| Modermate plolltion group | 544; 903; 1616; 1807; 1895; 1985; 1896; 2080-2090; 2094; 2095; 2102 |

Table S2. The position of rough characteristic bands based on the CWT method.

Note: soil samples were classified into three types based on the background value (BV) of the Inner Mongolia Autonomous Region using the contamination factor method, including clean (Zn ≤ 48.6 mg kg^-1^), low pollution (48.6 ≤ Zn ≤ 97.2 mg kg^-1^), and moderate pollution (97.2 ≤ Zn ≤ 145.8 mg kg^-1^), respectively. The rough characteristic bands were not observed in lab-based unprocessed and in-situ situations.
